# Trace Metal Enrichment and Radiological Risk in Coastal Sediments: Implications for Ecological and Human Health Safety

**DOI:** 10.3390/toxics14060464

**Published:** 2026-05-26

**Authors:** El Saeed R. Lasheen, Tamader Alhazani, Gehad M. Saleh, Basma A. El-Badry, Mabrouk Sami, Ioan V. Sanislav, Ahmed Abdelaal

**Affiliations:** 1 Geology Department, Faculty of Science, Al-Azhar University, Cairo 11884, Egypt; 2 Physics Department, Faculty of Science, Imam Mohammad Ibn Saud Islamic University (IMSIU), Riyadh 11564, Saudi Arabia; 3 Nuclear Materials Authority, Cairo 530, Egypt; 4 Geosciences Department, College of Science, United Arab Emirates University, Al Ain 15551, United Arab Emirates; 5 Economic Geology Research Centre (EGRU), College of Science and Engineering, James Cook University, Townsville, QLD 4811, Australia; 6 Environmental Sciences Department, Faculty of Science, Port Said University, Port Said 42522, Egypt

**Keywords:** Ras Mohamed sediments, metal pollution, radioactivity, geochemistry, South Sinai, Red Sea

## Abstract

Coastal environments are becoming more susceptible to enrichment of trace elements from human activities and natural processes. This research presents a detailed assessment of heavy metal pollution and radiological risks in coastal sediments from the Ras Mohamed area, South Sinai, at the northern Red Sea. Fifteen surface sediment samples were examined for nine trace metals and naturally occurring radionuclides (^226^Ra, ^232^Th, and ^40^K) using ICP-OES and gamma spectrometry techniques, respectively. Geochemical analyses showed the concentration sequence Fe > Ba > V > Cr > Zn > Co > Ni > Cu > Pb, where average levels of Cr, V, and Co were higher than Canadian Soil Quality Guidelines (CSQGs) and global crustal background values. Environmental evaluation using the pollution load index = 2.16 reflected ongoing contamination, and the Geo-Accumulation Index indicated low to moderate polluted sediment conditions. Nevertheless, ecological risk results (PERI = 87.21) together with toxicity indicators pointed to low to moderate biological effects. Human exposure assessments for adults and children revealed no significant non-carcinogenic risk (HI < 1), and the Total Cancer Risk remained below the acceptable regulatory threshold (1 × 10^−4^). From the other side, all recorded radiation activities were low, falling below internationally recognized safety limits. An evaluation of radiological hazard indices further confirmed that the sediments present no significant radiation risk, as all measurements remain within the low-level classification of international standards. Overall, the results indicate that although localized sediment transport and tourism-related pressures have increased certain metal levels, the region is radiologically secure and currently presents negligible risk to human health.

## 1. Introduction

Radioactivity is the natural and spontaneous transformation of an unstable atomic nucleus, occurring mainly due to naturally occurring sources: the long-lived primordial radionuclides uranium, thorium, and potassium [1,2,3,4]. These elements were formed by stellar nucleosynthesis and incorporated into the planet during its formation [5]. Their distribution within the crust is highly heterogeneous, resulting in significant variations in concentration across different rock types. For example, granitic rocks generally exhibit higher levels of radioactivity than most metamorphic rocks [6], a consequence of their enrichment in accessory minerals such as zircon [4,7]. The typical concentrations of U (ppm), K (%), and Th (ppm) in granitic rocks are 4, ~3–4%, and 17, respectively [8,9,10]. Although radioactivity is a natural phenomenon, human activities, including mining, nuclear power generation, and improper waste disposal, have significantly redistributed these materials in the environment. Its impacts are dual in nature: Radioactivity is critically beneficial in fields like medical imaging, cancer therapy, and archaeology [1,11,12,13], while prolonged exposure to elevated levels can lead to genetic mutations and an increased risk of cancer. Radionuclide pollution poses a significant threat to ecosystem integrity and human health through soil and water pathways. This makes the study of how these elements are distributed and move through the environment fundamental to radiation protection, environmental surveillance, and key geological fields like uranium prospecting and crustal studies [4,5,6,7,14,15,16,17,18].

**Figure 1:**
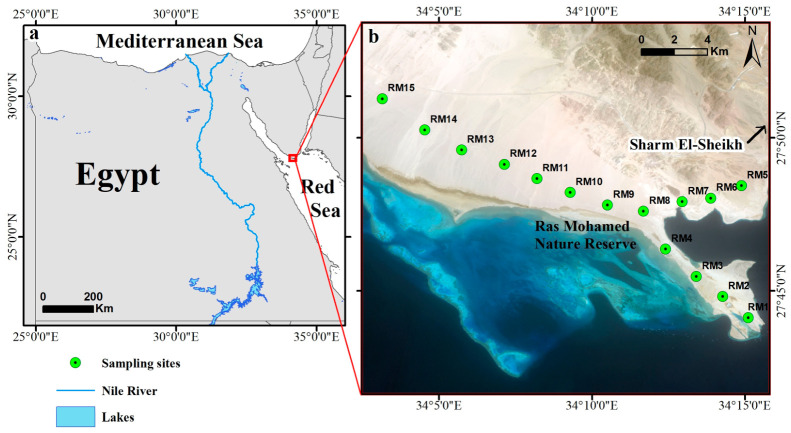
(**a**) Ras Mohamed area is in South Sinai, approximately 23 km south of Sharm El-Sheikh city, north Red Sea, South Sinai. (**b**) True-color Landsat-8 imagery (bands 4, 3, and 2) highlighting the study area’s surface features and the distribution of coastal sediment sampling locations.

The coastal zones of the Red Sea play a crucial role in the blue economy by sustaining economic activities such as tourism and mineral resource development (Figure 1a). These regions host extensive mangroves that offer critical ecological refuge and protection against erosion [19]. However, anthropogenic activities have increased sediment and metal pollution globally [20]. Such persistent contaminants pose significant risks to the ecosystem and human health via accumulation [21]. Consequently, continuous monitoring is essential to mitigate these impacts, particularly as erosion and deposition actively shape these coastal zones [22].

Expanding tourism along Egypt’s Red Sea threatens coral ecosystems [23]. Consequently, studies have analyzed coastal sediments to understand metal pollution distribution [24]. Identifying pollutant sources is crucial for management, prompting the use of various ecological and human health risk assessments, such as single-element indices (e.g., enrichment factor, contamination factor, and geo-accumulation index), integrated indices (pollution load index), and Sediment Quality Guidelines (SQGs) [19]. Regarding methodology, Hargalani [25] applied an innovative pollution index in the Caspian Sea to assess bioavailability and anthropogenic contributions. Similarly, Vaezi [26] utilized a new index for Mahshahr Bay, notably observing that conventional assessment techniques may be unsuitable for regions with high natural lithogenous backgrounds. Previous investigations along the wider Egyptian Red Sea coastline and the Gulf of Suez documented lower baseline levels of toxic trace metals, establishing a standard for regional environmental assessments [23,24]. Localized evaluations at Marsa Alam shoreline and Abu Minqar Island revealed varied heavy metal concentrations heavily dictated by near-shore sedimentation patterns and restricted coastal marine traffic. Studies targeting the pristine environments of Wadi El-Gemal Island and the El Qulaan archipelago identified minimal anthropogenic footprints, with heavy metal distributions remaining safely within natural background limits. Conversely, active transport studies across the mainland Wadi El-Gemal outlets demonstrated elevated heavy metal accumulation at wadi mouths, driven by the erosional land-to-sea transport of mineralized inland rocks [27].

This study comprehensively examines metal pollution in sediments of the Ras Mohamed area, northern Red Sea coast, South Sinai. It utilizes geochemical analyses, GIS mapping, and risk assessment indices to evaluate spatial patterns, ecological effects, and health hazards from cutaneous exposure. Furthermore, this study assesses sediment radioactive potential by measuring penetrating radiation dosages (^232^Th, ^226^Ra, and ^40^K) and calculating radiological risk indices, providing critical data for nuclear, construction, and tourism applications.

## 2. Study Area and Geology Setting

**Figure 2:**
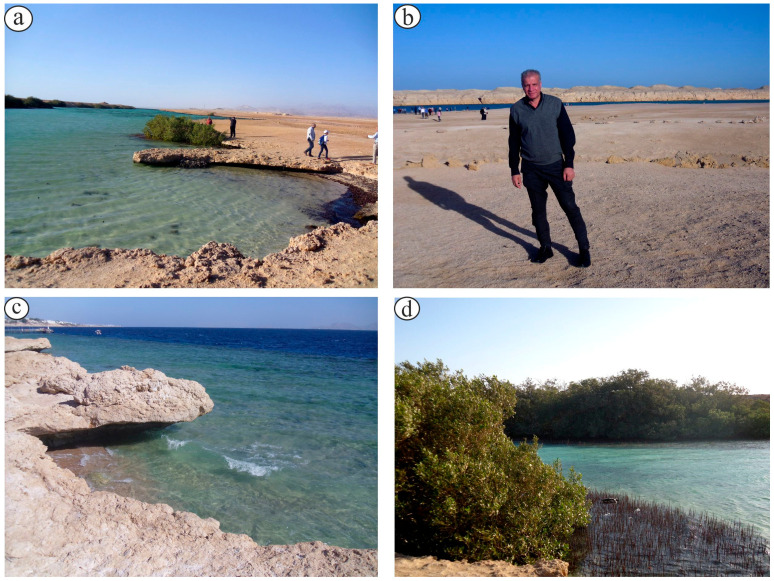
Field photographs of the Ras Mohamed area, north Red Sea, South Sinai. Quaternary reef limestone (**a**–**c**) and wide distribution of mangrove swamps (**d**). The person appears in this photo is the third author with no copyright issue, who supported sample collection and the field trip.

The Ras Mohamed area, situated in South Sinai, 23 km south of Sharm El-Sheikh city (Figure 1b), is part of the Egyptian Nubian Shield [28]. The geology of the Ras Mohamed area in southern Sinai occupies a tectonically dynamic setting at the confluence of three major rifts, resulting in intense faulting. The region features both crystalline Proterozoic basement rocks and widespread sedimentary cover [29,30]. The Ras Mohamed area is characterized by two major Neogene blocks partly covered by Quaternary reef limestone. The area features prominent raised coral reefs, some reaching 30 m in height, which define much of the landscape (Figure 2a–c). Notable geological formations also include an earthquake crack that hosts anchialine pools (landlocked saline water bodies). Tectonic activity has generated an earthquake fissure, which forms a series of inland anchialine pools. These tidal pools are flooded by seawater and fluctuate with the tides. The area is dissected by a prominent fault network with dominant NW–SE and NE–SW trends. A Landsat-8 OLI satellite image (Path: 174; Row: 41), acquired on 14 August 2025, from the USGS (https://earthexplorer.usgs.gov/, accessed on 21 May 2026), was used to delineate the sediment sites and land use features in the Ras Mohamed area (Figure 1b). A true-color composite (bands 4, 3, 2) was generated to delineate land use characteristics [31]. Geologically, Quaternary sediments of the Red Sea are distributed along the coast, running parallel to the Nubian crystalline rocks. Furthermore, mangrove swamps are present (Figure 2d); their dense branching structures produce significant organic material. This detritus is exported to surrounding areas, enhancing the overall productivity and ecological function of the mangrove ecosystem.

## 3. Materials and Methods

### 3.1. Radioactive Detection

**Figure 3:**
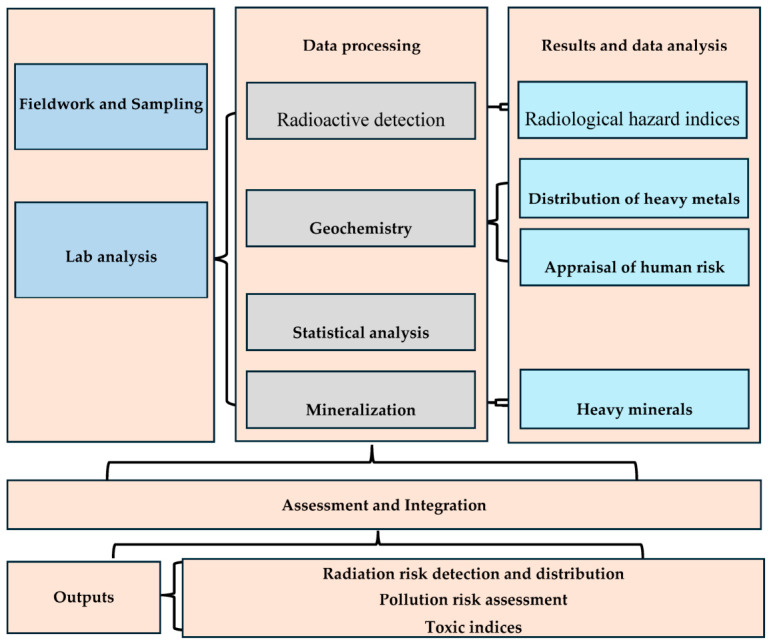
Schematic diagram of research methodology for the Ras Mohamed area.

Gamma-ray spectrometry was employed to detect radionuclide activity levels (Figure 3). Prior to measurement, fifteen sediment samples (~350 g each) from Ras Mohamed were sealed and stored for a minimum of 25 days to establish radioactive equilibrium. Analysis was conducted at the Nuclear (NMA) Material Authority (Cairo, Egypt) using a Bicron NaI (Tl) detector (76 × 76 mm scintillation crystal), specifically measuring the 1460.8 keV gamma peak for ^40^K and the 238.6 keV peak (from ^212^Pb) for ^232^Th. Comprehensive details on energy calibration, uncertainties, and detection limits are documented in the Supplementary Materials. To evaluate the potential health risks from radiation, a series of standardized hazard parameters was estimated using the measured activity levels of ^232^Th (Y_Th_), ^226^Ra (Y_Ra_), and ^40^K (Y_K_) (Table 1). The derived indicators included the following: absorbed dose rate in air (D_air_), radium equivalent activity (Ra_eq_), external and internal hazard indices (H_ex_ and H_in_), gamma and alpha indices (I_γ_ and I_α_), annual effective dose for outdoor and indoor (AED_out_ and AED_in_), and excess lifetime cancer risk (ELCR) (Table 1). All indicators were computed using established protocols.

### 3.2. Geochemistry

#### 3.2.1. Sediment Sampling and Analysis

Surface sediment samples (S1–S15) were collected from the top layer (0–10 cm) of the Ras Mohamed coastal zone, South Sinai, using a manual auger (Figure 1b). The fifteen sampling points across the Ras Mohamed coastal zone were carefully selected based on the geological maps, Landsat-8 satellite spatial grid, and field investigation to capture the transition between the Quaternary sediments and the mineralized Nubian crystalline basement rocks. The locations were specifically aligned with the mouths of major wadi channels to intercept heavy metals transported from inland alteration zones during natural flash floods. Furthermore, the network targeted sensitive mangrove swamps to monitor organic matter content and chemical baseline fluctuations. Ultimately, this layout guarantees an accurate representation of the coast by pairing these natural drainage zones with localized areas subjected to intensive marine tourism and regional mining footprints. Samples were collected in clean 1 kg bags and transported to the laboratory for geochemical analysis (Figure 3), including pH and total organic matter (TOM) measurements. Grain size analysis was performed on about 30 g of each sample. To eliminate carbonates and organic debris, samples were treated with 15% H_2_O_2_ and diluted HCl [34]. Textural classification was examined using ternary diagrams [35], facilitating identification based on grain size: gravel, sand, or mud (silt and clay) [36]. Sand and gravel fractions were determined via wet sieving, while the mud fraction utilized the pipette method strictly [35]. Organic matter content (2 g samples) was quantified by loss-on-ignition at 550 °C [37]. Materials were re-weighed to calculate the percentage weight excluding organic components. The finest sediment fractions (<63 µm) were utilized for metal analysis. These were prepared, involving grinding, screening, and air-drying at room temperature. Samples (1 g) were digested using a nitric, perchloric, and hydrochloric acid mixture and then filtered to remove residuals [38]. Nine metals were analyzed using ICP-OES at the NMA. Detection limits for Ba, Co, Pb, Cu, Cr, Ni, Zn, V, and Fe were 0.2, 0.2, 1, 1, 0.5, 1, 3, 0.1, and 10 mg/kg, respectively. Accuracy was verified using NIST (Boulder City, Colorado, USA) and Merck (Darmstadt City, Germany) reference standards, with recovery rates ranging from 92% to 101%.

#### 3.2.2. Ecological, Sediment Quality Guidelines (SQGs), and Health Risk Assessment Indices

This study calculated and applied five ecological risk indices (Figure 3). The Enrichment Factor (EF) [39] was determined as EF = (C_s_/Fe_s_)/(C_b_/Fe_b_), where Cs represents the sample’s metal concentration, C_b_ is the crustal background, and Fe_s_ and Fe_b_ serve as iron normalization references. The Earth’s crust background levels for Ba, Co, Pb, Cu, Cr, Ni, Zn, V, and Fe were established at 668, 11.6, 17, 14.3, 35, 18.6, 52, 60, and 30,890 mg/kg, respectively [40]. The contamination factor (CF) was calculated as CF = C_s_/C_b_ [41], representing the ratio of detected metal concentrations (C_s_) to local reference values (C_b_) [42]. The index of geo-accumulation (I_geo_) relates the investigated metal value (C_n_) to the crust-background (B_n_), applying a normalizing factor of 1.5 [42] and calculated as $Igeo={log}_{2}﴾\frac{Cn}{1.5*Bn}﴿$ (Table S1). The pollution load index (PLI) [43] was derived using PLI = (CF1 * CF2 * CF3 * CFn)^1/n^, where n denotes the nine analyzed metals and CF represents their respective contamination factors. The Potential Ecological Risk Index (PERI) was calculated by summing the potential ecological risk factors $\sum_{i=1}^{n}Eri=\;\sum_{i=1}^{n}Tri\times CFi$, utilizing specific toxic response factors (Tr^i^) for Co (5), Pb (5), Cu (5), Cr (2), Ni (5), Zn (1), V (2), and Fe (1) [41]. Additionally, three SQGs were employed. The Mean Effects Range Median Quotient (MERMQ) was calculated as MERMQ = $\frac{\sum \left(Ci/ERMi\right)}{n}$, relating measured metal values (C^i^) to the Effects Range Median (ERM^i^) [44]. The Toxic Risk Index (TRI) was computed as TRI^i^ = $\sqrt{\frac{\left(\frac{Ci}{TEL}\right)2+\left(\frac{Ci}{PEL}\right)2}{2}}$ and TRI = $\sum_{i=1}^{n}TRIi$ for both individual elements (TRI^i^) and integrated metals based on SQG Threshold Effects Levels (TELs) and Probable Effects Levels (PELs) [45]. The modified Hazard Quotient (mHQ) was calculated as mHQ = $\sqrt{\frac{Ci}{TELi}+\frac{Ci}{PELi}+\;\frac{Ci}{SELi}}$ by assessing metal concentrations against TEL, PEL, and Severe Effect Level (SEL) standards [46]. Furthermore, two human health risk indices were assessed: the non-carcinogenic hazard index (HI) calculated as HI = $\sum_{i=1}^{n}HQi$ [47,48], where HQ = CDI/RfD and CDI_oral_ = (Cs × CF × SA × AF × ABS × EF × ED)/(BW × AT), which incorporates surface area, adherence, and exposure factors (Tables S1 and S2). The Total Cancer Risk (TCR) was calculated as TCR = ∑CR and CR = CDI_oral_ × CSF, applying Cancer Slope Factors (CSFs) of 0.0085, 0.5, and 0.091 for Pb, Cr, and Ni, respectively. Detailed input data and equations for all ecological, SQG, and health risk indices are provided in the Supplementary Materials.

#### 3.2.3. Mineralization

To assess the potential presence of radioactive minerals in the coastal sediments, the collected material was initially air-dried and split into subsamples. A fraction of these underwent density separation using bromoform to isolate the heavy mineral concentrate. This concentrate was then further separated by magnetic susceptibility via a Frantz isodynamic separator. At the NMA laboratories, specific heavy mineral grains were manually selected under a binocular microscope, and their identities were confirmed using an Environmental Scanning Electron Microscope (ESEM; Figure 3).

#### 3.2.4. Statistical Analysis

To evaluate the potential origins of metal contaminants, statistical analyses, including summary statistics (Figure 3), Pearson correlation, and Principal Component Analysis (PCA), were conducted using Statgraphics (v. 18) software [49]. The Kolmogorov–Smirnov test was employed to verify the normality of the pH, organic matter, and metal concentration datasets. Skewness and kurtosis were utilized to assess the grain size distribution. The results indicated that all parameters followed a normal distribution (Table 2).

## 4. Results and Discussion

### 4.1. Sediment’s Composition and Type

**Figure 4:**
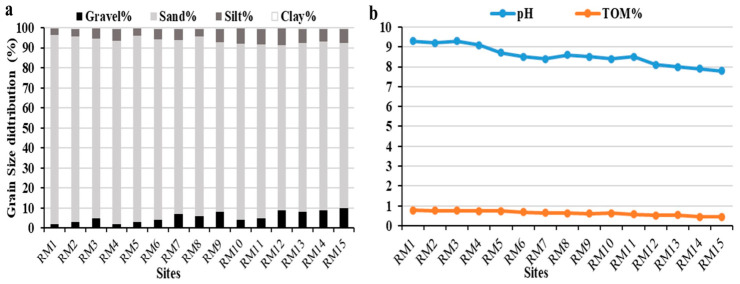
Spatial patterns of sediment grain size (**a**) alongside pH and total organic matter (TOM%; (**b**)) in coastal sediments of the Ras Mohamed region, South Sinai.

Table S3 reports the investigated values for pH, total organic matter (TOM), and sediment grain size and type, Table 2 summarizes the results, and Figure 4 shows their spatially distributed values. The findings indicated that the grain size distribution of Ras Mohamed coastal sediments shows the relative proportions of sediment fractions. Sand (light gray) is the dominant component across all sites, typically accounting for over 80–90% of the distribution. Gravel (black) and silt (dark gray) constitute minor portions of the sediment, while clay levels are negligible (<0.5%). A slight increase in the gravel fraction is observable in the later sites (RM12–RM15) (Figure 4a). The sediments exhibit alkaline conditions, with pH values (blue line) peaking at approximately 9.3 at RM1 and gradually decreasing to about 7.8 at RM15. In contrast, TOM% (orange line) remains consistently low, staying below 1% across all sampling locations [51]. It shows a very slight downward trend from the first to the last site, paralleling the pH gradient (Figure 4b). The distribution of grain size, pH, and organic matter in Ras Mohamed sediments is shaped by both natural processes and anthropogenic impacts. The presence of Quaternary Red Sea sediments running parallel to Nubian crystalline rocks along the coastline serves as a primary source for the dominant sand and gravel fractions. The spatial distribution of these particles is shaped by natural processes of erosion and deposition occurring along the Red Sea coastal zones and islands. The abundant mangrove populations in these productive marine ecosystems play a critical role in nutrient cycling and sediment stability, which can influence local organic matter levels (Figure 3). Increasing human activities, such as urban expansion, industrial operations, and agricultural practices, contribute to the influx of nutrients and sediments. The expanding tourism sector along the Red Sea coastline can introduce external pollutants and organic materials that alter the natural chemical balance of the coral reef ecosystem.

### 4.2. Metal Contents and Spatial Pattern

Table 2 summarizes metal contents in coastal sediments of the Ras Mohamed area (n = 15), which are reported in Table S4. The recorded mean concentrations (mg/kg) follow a descending order: Fe (67.347) > Ba (213.13) > V (201.53) > Cr (136.67) > Zn (87.93) > Co (67.53) > Ni (48.47) > Cu (47) > Pb (34.87). The concentrations of most metals, particularly Pb, Cr, Ni, and V, significantly exceed Earth’s crustal background levels [40]. Specifically, Cr (136.67), V (201.53), and Co (67.53) also surpass the Canadian Soil Quality Guidelines [50]. This enrichment is related to the strategic location of Ras Mohamed’s mangrove sites at the terminus of the wadis to the west. These wadis drain alteration zones rich in mineral deposits like copper, lead, and iron, transporting them downstream during flood events [19,27]. Other contributing factors include the weathering of basement rocks, mining operations, and intensive tourism activities along the Red Sea shoreline [24]. Globally, anthropogenic footprints in coastal regions are primarily driven by intensive mining operations, energy generation, and improper municipal or industrial waste disposal, which redistribute persistent trace metals and radionuclides into marine zones. In the specific context of the Ras Mohamed area, the impact of human activity manifests distinctly as localized tourism-related pressures and minor urban expansions along the northern Red Sea coastline. Intensive coastal tourism activities near the northern mangrove beaches are directly linked to elevated levels of Co and V in the sediments. Furthermore, regional mining operations and the weathering of heavy-metal-rich basement rocks add to the influx of pollutants, contributing to the selective accumulation of Pb, Ni, and Zn in the western sectors of the study area. While natural wadi flash floods remain a primary pathway for transporting mineralized materials downstream into the coast, expanding marine tourism represents the most immediate, escalating human threats to the chemical balance of this protected coral ecosystem. Despite these elevations, the concentrations of Co, Cu, Zn, and Fe remain within the safety limits [50]. Conversely, almost all metals exceed the UCC background values [40], highlighting a distinct accumulation relative to average crustal composition.

**Figure 5:**
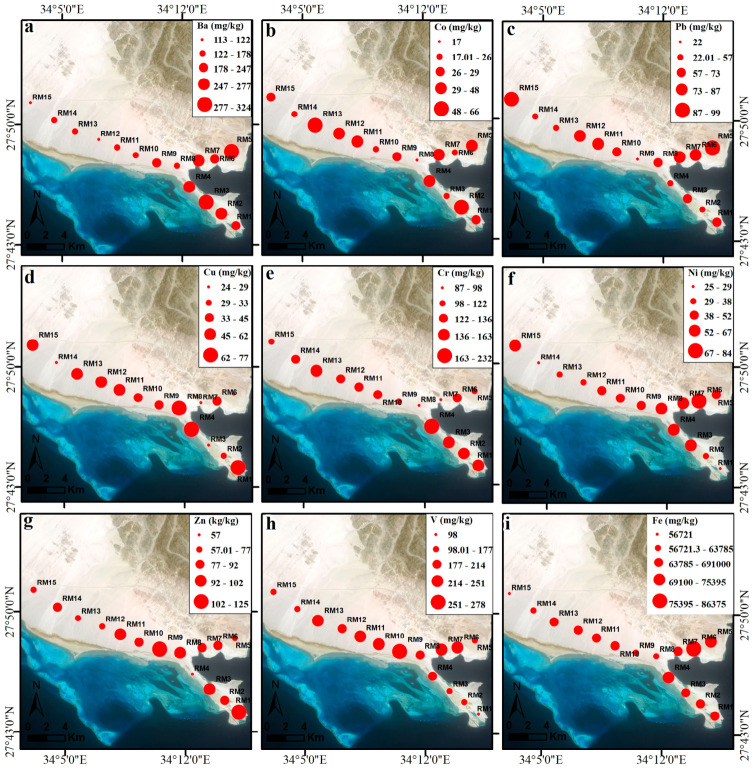
Spatial pattern of metal contents (mg/kg) in Ras Mohamed coastal sediments, South Sinai: Ba (**a**), Co (**b**), Pb (**c**), Cu (**d**), Cr (**e**), Ni (**f**), Zn (**g**), V (**h**), and Fe (**i**).

Distributions of examined metal concentrations across Ras Mohamed sediments (Figure 5) reveal that northern sites (RM1, RM6–RM7) exhibit high levels of Co (Figure 5b) and V (Figure 5h) linked to tourism activities near the northern mangrove beach. The central and southeastern locations (RM2–RM5 and RM8–RM10) show peak concentrations for most metals, including Ba (Figure 5a), Cu (Figure 5d), Cr (Figure 5e), and Fe (Figure 5i). This enrichment likely originates from wadi-rich materials discharged from mineralization zones to the west, which transport minerals to the coast during flood events. Furthermore, western sites (RM11–RM15) display elevated levels of Pb (Figure 5c), Ni (Figure 5f), and Zn (Figure 5g), potentially influenced by basement rock weathering and regional mining operations [24]. Anthropogenic contamination in the Ras Mohamed region is primarily driven by expanding tourism sectors and regional mining operations along the northern Red Sea coastline [19]. Intensive marine tourism and localized infrastructure pressures near the northern mangrove beaches directly introduce external pollutants, driving localized elevations of Co and V [19]. Simultaneously, regional mining activities and human-induced land modifications aggravate the redistribution and influx of trace metals into the marine environment. These combined industrial and recreational developments supplement natural weathering, leading to the selective accumulation of toxic elements like Pb, Ni, and Zn in the western sectors of the study area. Consequently, these localized human activities present an escalating threat that disrupts the baseline geochemistry of this protected coral ecosystem [19,24,52]. Table 3 provides a comparative assessment of mean metal concentrations in Ras Mohamed sediments against various regional and international datasets. The average concentrations of Ba, Pb, Cr, Ni, and V at Ras Mohamed significantly exceed those reported for the Egyptian Red Sea coastline [53], the W. El-Gemal outlet [24], and W. El-Gemal Island [54]. Similarly, these levels surpass those found at Abu Minqar Island, Abu Ghusun, and El Qulaan [23,55]; Marsa Alam [56]; the Gulf of Suez [57]; and across the border at Ras Abu Ali Island and the Aqaba coast in Saudi Arabia [58]. Higher values were also noted compared to Mahshahr Bay and the Northeast coast of Iran [20], as well as lagoon-lake sediments in Turkey [59]. Conversely, the average Co, Cu, Zn, and Fe contents in the study area are lower than those recorded in coastal sediments from Egypt, China, India, and Taiwan [22,55,60,61]. Spatial analysis further illustrates these trends, with localized peaks for metals like Pb, V, and Ni occurring at specific sampling sites such as RM1, RM6, and RM15 (Figure 5). The recorded average concentrations of barium, lead, chromium, nickel, and vanadium in Ras Mohamed coastal sediments significantly surpass those reported for the broader Egyptian Red Sea coastline, the Gulf of Suez, and localized coastal regions such as Marsa Alam, the Wadi El-Gemal outlets, and Abu Minqar Island. This enrichment also extends globally, with these key trace metals exceeding concentrations observed along the Saudi Arabian Aqaba coast, Ras Abu Ali Island, Mahshahr Bay in Iran, and lagoonal environments in Turkey. Specifically, the mean levels of chromium (136.67 mg/kg), vanadium (201.53 mg/kg), and cobalt (67.53 mg/kg) explicitly outstrip both average global crustal backgrounds and the Canadian Soil Quality Guidelines. Conversely, elements such as copper, zinc, and iron remain comparatively lower than benchmarks from heavily industrialized regions of China, India, and Taiwan (Table 3). This specific contamination pattern points to a distinct combination of natural inputs via mineralized wadi flash floods alongside localized anthropogenic pressures, including expanding regional mining operations and intensive marine tourism footprints along the shoreline.

### 4.3. Potential Source of Metal Pollution

**Figure 6:**
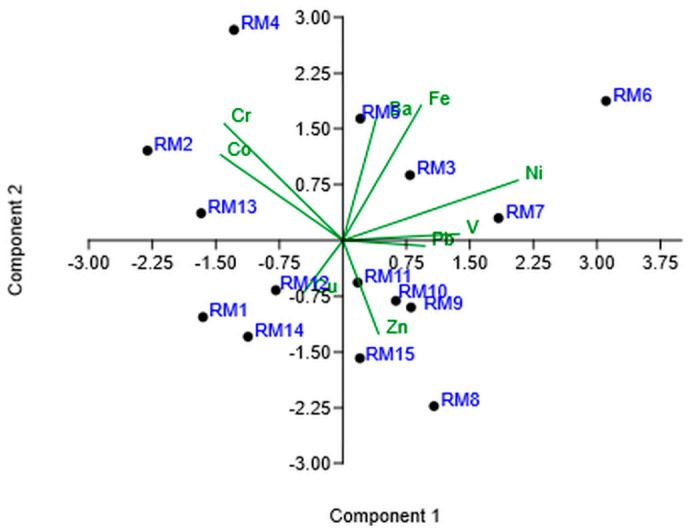
PCA plot of the studied metals in Ras Mohamed sediments, South Sinai.

PCA was used to explore hidden associations among variables and to better characterize the multidimensional structure of the data. The analysis identified five principal components for metals in Ras Mohamed coastal sediments (Table 4). The first component (PC1) accounted for 24.09% of the total variance, with strong positive loadings for Ni (0.58) and V (0.39) (Figure 6), suggesting a shared geochemical behavior; while these elements are sometimes linked to fuel combustion, their regional presence often relates to natural crustal weathering. The second factor (PC2) explained 23.08% of data variance with significant and positive loadings of Ba (0.47), Co (0.32), Cr (0.44), and Fe (0.51) (Table 4 and Figure 6). This usually suggests a common lithogenic source inherent to the local geology. Previous research indicates that certain metals exhibit positive correlations, implying similar sources [22,26,61,63]. The third factor (PC3) accounted for 14.91% of the overall data variation, with significant loading of Zn (−0.62) and Pb (0.42) (Figure 6). Their opposing loadings suggest distinct environmental signatures, potentially reflecting localized coastal influences that require further validation. PC4 (14.17% variance) is dominated by Cu, while PC5 (11.83% variance) reveals a secondary, independent behavior for V (Figure 6). Given our limited sample size, these associations are treated as exploratory indicators of geochemical clustering [62]. Further high-resolution spatial and temporal monitoring is recommended to substantiate these preliminary hypotheses.

### 4.4. Ecological Risk Assessment Indices

**Figure 7:**
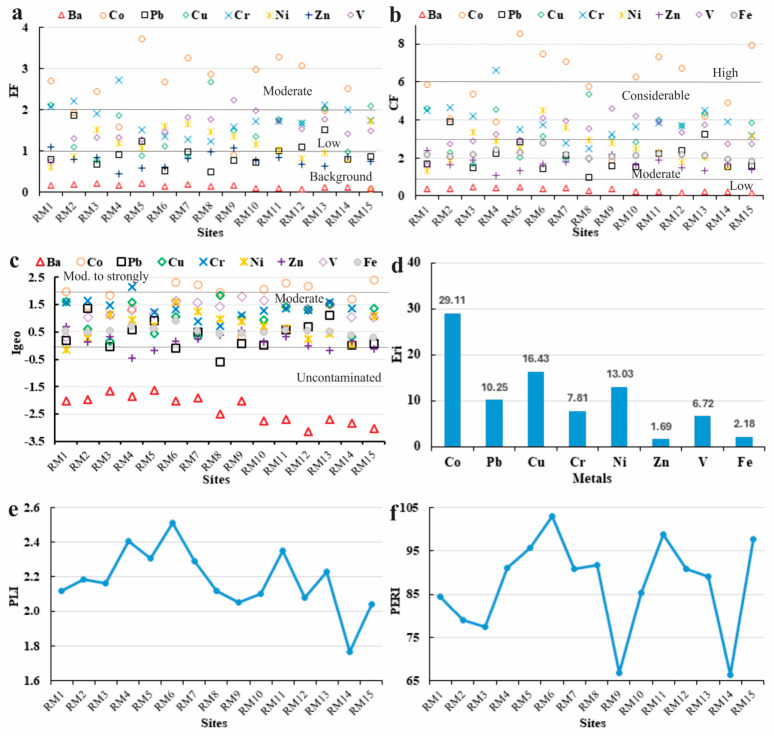
Assessment results of EF (**a**), CF (**b**), I_geo_ (**c**), Eri (**d**), PLI (**e**), and PERI (**f**) for the investigated metals in Ras Mohamed sediments, South Sinai.

Table S5 and Figure 7a present the EF of the metals investigated in sediments of the Ras Mohamed area, South Sinai. The data indicate that the mean EFs for all examined metals reflect varying degrees of environmental accumulation. Specifically, the mean EFs for Co (2.35), Cu (1.49 and 1.77 for both columns), and V (1.56) suggest categories ranging from minimal to moderate enrichment. While metals like Ba (0.15) and Pb (0.96) show average EFs indicative of deficiency to minimal enrichment, Ni (1.13) and Zn (0.82) also fall within the minimal enrichment category (EF < 2). These variations in enrichment levels are consistent with findings from regional studies, highlighting the influence of both natural mineral transport and anthropogenic activities on sediment quality [62,63,64].

The contamination factor (CF) calculated for metals in Ras Mohamed sediments is detailed in Table S6 and Figure 7b. The mean CF values across the study area follow a descending order: Co (5.83) > V (3.30) > Cr (3.89) > Cu (3.30) > Ni (2.59) > Fe (2.16) > Pb (2.05) > Zn (1.67) > Ba (0.32). Cobalt (Co) exhibited the highest average CF (5.83), with values at sites like RM5 (8.53), RM15 (7.93), and RM6 (7.50) indicating high contamination (CF ≥ 6). Significant contamination (3 ≤ CF < 6) was also observed for V, Cr, and Cu, particularly at sites such as RM9 (V = 4.63) and RM4 (Cr = 6.63), which may be linked to mineralized wadi-rich materials discharged from basement rock mining sites to the west (Figure 1b). Furthermore, Ni, Fe, Pb, and Zn displayed moderate contamination (1 ≤ CF < 3), suggesting that tourism activities along the beachfront and natural geological weathering contribute to the sediment’s metal load. In contrast, Ba showed low contamination (CF < 1) across all sampling sites, ranging from 0.17 to 0.49. These findings align with regional assessments of sediment quality under combined natural and anthropogenic pressures [41,55].

The ecological geo-accumulation index (I_geo_) for investigated metals in Ras Mohamed’s coastal sediments is detailed in Table S7 and Figure 7c. The mean I_geo_ values across the study area follow a decreasing order: Co (1.87) > Cr (1.34) > V (1.30) > Cu (8.70) > Ni (0.72) > Fe (0.53) > Pb (0.36) > Zn (0.14) > Ba (−2.31). Individual I_geo_ readings for all examined metals ranged from −3.15 (Ba at RM12) to 2.51 (Co at RM5), with an average value 0.56 across all elements (Figure 7c). According to the classification by [65], these results indicate that the analyzed sediments are low to moderately contaminated, as they fall into Class 1 to 3 (2 ≤ I_geo_ > 3). Specifically, Co shows the highest accumulation levels across all sites, followed by V, Cr, and Cu (1 < I_geo_ ≤ 2), while metals such as Zn, Pb, Fe, and Ni exhibit low to moderate enrichment (0 < I_geo_ ≤ 1), and Ba shows uncontaminated sediments throughout the investigated coastal area.

We acknowledge that the Ras Mohamed area is geochemically distinct due to carbonate sedimentation and basement rock weathering. As local background reference data for this specific coastal environment is currently absent, we utilized global Upper Continental Crust (UCC) values [40], a standard approach widely adopted in international geochemical research. We explicitly discuss this methodological choice as a necessary standardization that, while robust, introduces unavoidable uncertainty regarding absolute enrichment estimates. By framing our results against these recognized global benchmarks, we provide a conservative and transparent assessment of environmental quality. This clarification reinforces our findings while rigorously managing the limitations inherent in baseline selection for unique geological settings.

Table S6 illustrates the pollution load index (PLI) values for metals investigated in Ras Mohamed sediments. The calculated average PLI value of 2.16, with a range from 1.76 to 2.51, indicates progressive contamination (PLI > 1) across all sampling sites (Figure 7e). The PLI data suggest that the discharge of mineralized materials from the wadis to the west of the study area, coupled with local tourism and basement rock weathering, has led to significantly polluted sediments [41,55]. To assess the potential toxicity and ecological impact of metal concentrations in Ras Mohamed sediments, researchers employed the potential ecological risk index (PERI) and the individual potential ecological risk factor (Er^i^). The specific findings for each sampling site and metal are detailed in Table S8 and Figure 7f. The mean Er^i^ values for the investigated metals followed a descending order: Co (29.11) > Cu (16.43) > Ni (13.03) > Pb (10.25) > Cr (7.81) > V (6.72) > Fe (2.18) > Zn (1.69) (Figure 7d). According to the classification established by [46,60,64], all analyzed metals were categorized as low risk (Er^i^ < 40) across all sampling sites (Figure 7d). Furthermore, the cumulative PERI values for the study area ranged from 66.36 to 103.05, averaging 87.21 (Figure 7f). These results indicate that the investigated area remains within the low-risk grade (PERI < 150), despite the elevated geo-accumulation and contamination factors observed for specific elements [22,62]. These low-risk classifications arise because the Er^i^ and PERI indices are fundamentally designed to integrate both concentration and specific toxic response factors (Tr^i^), which effectively normalize the impact of each element relative to its biological toxicity. Although individual elements like Co and Cu display higher enrichment factors, their absolute concentrations, when weighted by their assigned toxic response, do not reach the threshold necessary to trigger high-level ecological alerts. This divergence suggests that while specific industrial or geological factors influence metal enrichment, the overall sediment quality in the Ras Mohamed reserve maintains an ecological balance that does not currently pose a critical threat to local marine biota.

### 4.5. Sediment Quality Guidelines (SQGs)

**Figure 8:**
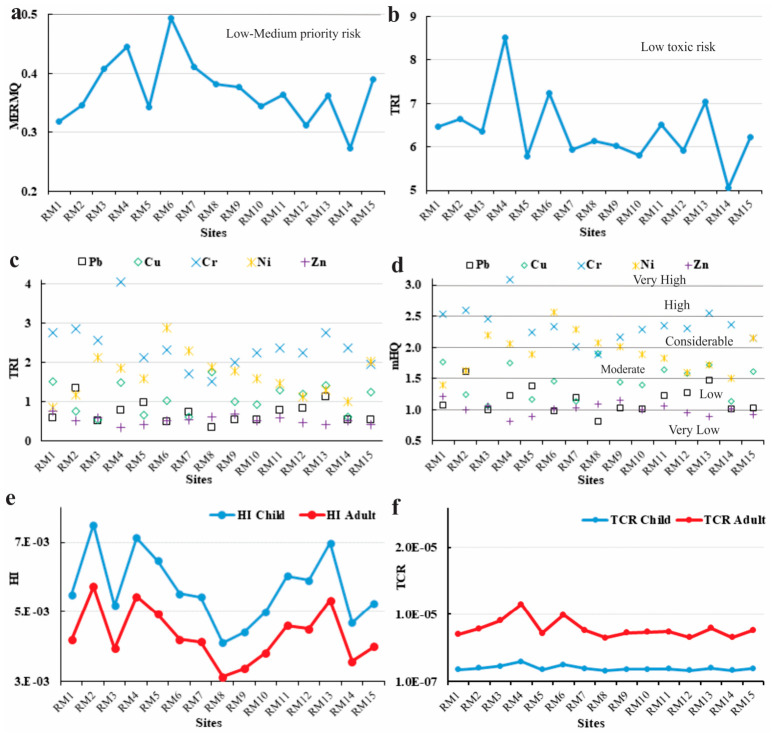
Calculated SQG values including MERMQ (**a**), TRI (**b**,**c**), mHQ (**d**), HI (**e**), and TCR (**f**) for the investigated metals in Ras Mohamed sediments, South Sinai.

The calculated mean effects range median quotient (MERMQ) values for the metals examined in Ras Mohamed sediments are presented in Table S9 and Figure 8a based on the effects range low (ERL) and effects range median (ERM) criteria [44]. The table displays the comparison results of the SQGs ERL, ERM, threshold effect level (TEL), and probable effect level (PEL) against the Pb, Cu, Cr, Ni, and Zn contents recorded in the study area. The computed MERMQ values for these metals across the 15 sampling sites ranged from 0.27 to 0.49, with a calculated average value of 0.37. According to established risk categories, these MERMQ values represent a low-priority risk level (MERMQ ≤ 0.1 being very low and 0.1 < MERMQ ≤ 0.5 being low), suggesting approximately a 21% probability of toxicity to benthic organisms [44]. Specifically, site RM6 showed the highest MERMQ (0.49), primarily driven by elevated Ni levels, while site RM14 exhibited the lowest quotient (0.27). These findings indicate that while metals are present, the current concentrations in the Ras Mohamed sediments are generally below the thresholds expected to cause frequent adverse biological effects [44]. Table S10 and Figure 8b,c present the calculated Toxic Risk Index (TRI) values for Pb, Cu, Cr, Ni, and Zn in the sediments of the Ras Mohamed area, derived from the SQG, TEL, and PEL benchmarks. The multi-element TRI values across the fifteen sampling sites varied from 5.06 to 8.53, with an average of 6.39. According to the established classification, these results indicate low toxic risk (TRI ≤ 10) for the coastal area. Among the individual metals, Cr (mean TRI = 2.91) and Ni (mean TRI = 2.85) exhibited the highest average toxic risk contributions, while Pb (mean TRI = 2.06), Cu (mean TRI = 0.81), and Zn (mean TRI = 0.58) showed significantly lower values. Specifically, the highest localized toxic risk was observed at site RM4 (8.53), followed by RM6 (7.24), primarily due to elevated levels of Cr and Ni, whereas site RM14 (5.06) presented the lowest risk [39] (Figure 8c).

The mHQ values for the examined metals in Ras Mohamed coastal sediments, calculated based on SQG thresholds (TEL, PEL, and severe effect level (SEL)), are given in Table S11 and Figure 8d. Mean mHQ values varied between 0.82 and 3.09, with an overall average of 1.64, reflecting moderate contamination (1.5 ≤ mHQ < 2.0). The metals’ average mHQ values are ranked in a descending sequence: Cr (2.36) > Ni (1.91) > Cu (1.49) > Pb (1.16) > Zn (1.02). Furthermore, Cr exhibited the highest contamination severity, with values reaching 3.09 at site RM4, indicating high contamination severity (mHQ ≥ 2.5). Ni and Cu demonstrated moderate severity, while Pb and Zn generally showed low contamination severity (1.0 ≤ mHQ < 1.5) [44,45].

### 4.6. Human Health Risk Assessment

The non-carcinogenic Hazard Index (HI) values for metals in Ras Mohamed sediments, South Sinai, are presented in Table S12 and Figure 8e. HI levels for both adults and children were estimated using hazard quotients (HQs), based on the chronic daily intake and reference dose for specific exposure pathways. According to Table S12, the hazard quotients for individual metals across all sampling sites remain well below the allowable limit (HQ < 1). Specifically, the cumulative HI_Child_ values ranged from 4.12 × 10^−3^ (RM8) to 7.51 × 10^−3^ (RM2), with a calculated mean of approximately 5.63 × 10^−3^. The HI_Adult_ values were slightly lower, ranging from 3.14 × 10^−3^ (RM8) to 5.73 × 10^−3^ (RM2), with an average level of 4.31 × 10^−3^. The Cr and Pb contributed the highest quotients to the overall risk in both groups, yet their individual values did not pose a health threat. Consequently, the HI values indicate that there is no chronic non-carcinogenic risk for either children or adults from the sediments in the Ras Mohamed area, as all values remain significantly below the safety threshold (HI < 1) [48,62].

Table S13 and Figure 8f present the Total Cancer Risk (TCR) estimates for children and adults in Ras Mohamed sediments, with TCR for Pb, Cr, and Ni derived from cancer risk (CR), cancer slope factor (CSF), and chronic daily intake (CDI). According to the data, Cr exhibited the highest individual CR levels for both children (2.14 × 10^−6^ at RM4) and adults (8.15 × 10^−6^ at RM4), whereas Pb and Ni generally showed much lower values. Additionally, TCR_Child_ levels for the combined metals in the sediments under study ranged from 2.66 × 10^−9^ to 2.14 × 10^−6^, with a mean level of 6.92 × 10^−7^. In contrast, TCR_Adult_ values were higher, ranging from 1.02 × 10^−8^ to 8.15 × 10^−6^ with a mean value of 2.64 × 10^−6^. Table S13 and Figure 8f demonstrate that the TCR values of Pb, Cr, and Ni in the sediments of Ras Mohamed were obviously below the acceptable regulatory thresholds, which are 1 × 10^−6^ to 1 × 10^−4^ for multi-element risk [48,62]. Assessing TCR is crucial as long-term exposure to trace elements like Pb, Cr, and Ni can result in various cancers [66].

### 4.7. Radionuclide Abundance

**Figure 9:**
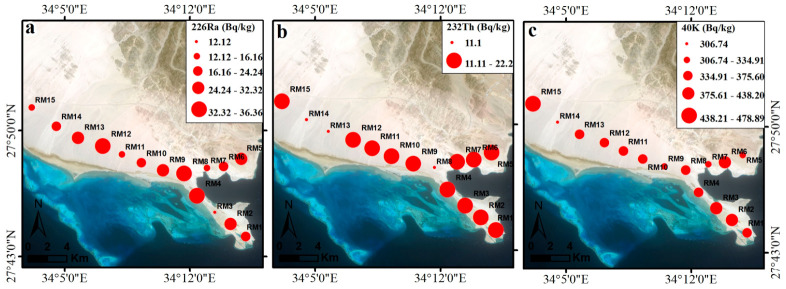
Spatial distribution of the radionuclides (Bq/kg) in sediments of the Ras Mohamed area, South Sinai: ^226^Ra (**a**), ^232^Th (**b**), and ^40^K (**c**).

Analyses using NaI(Tl) spectrometry on fifteen samples measured the activity levels of ^40^K, ^232^Th, and ^226^Ra (Table 5 and Figure 9). The mean values of 19.98 ± 4.44 Bq/kg for ^226^Ra (Figure 9a), 25.32 ± 8.28 Bq/kg for ^232^Th (Figure 9b), and 368.69 ± 44.52 Bq/kg for ^40^K (Figure 9c) are comparatively low relative to recognized global averages [1,6,15,67,68,69]. The total activity across samples ranges from 338.04 to 517.25 Bq/kg, with a mean of 414 ± 43.69 Bq/kg, also below the established global limit of 420 Bq/kg [17,36,70,71]. The samples consistently follow the following activity concentration order: ^40^K > ^232^Th > ^226^Ra. The elevated ^40^K levels are likely due to leached feldspar derived from host rocks, particularly granitic sources [11,72,73,74]. The silica content of the samples also contributes somewhat to the availability of ^40^K. The relatively low activity concentrations observed in the samples could be explained by either a lack of nearby radiogenic source rocks, like granite, or by the sediments originating from parent materials that are inherently low in these radionuclides [62,75]. Furthermore, as shown in Table 5, the activity ratios in the Ras Mohamed sediments consistently fall below established global averages. The ratios for ^232^Th/^40^K (range: 0.03–1.0; average: 0.07), ^226^Ra/^40^K (range: 0.03–0.07; average: 0.05), and ^226^Ra/^232^Th (range: 0.31–1.83; average: 0.91) are all lower than the respective global reference values of 0.07, 0.07, and 1 [1,33,76].

The estimated activities, which are low (Table 6), align with the UNSCAR [33] global average, others from Egypt {e.g., Sharm El Luli [77], Ghadir sediments [78], Qulaan sediments [62], and Abu Ghusun sediments [55]}, and worldwide concentrations such as those of Saudi Arabia [79], Aegean sediments [2], Thailand sediments [80], Konya sediments [68], Jeddah soils [12], the Dois Rios coast [16], and Leepa Valley [81].

### 4.8. Radiation Hazard Impact

The radiological hazard assessment for the Ras Mohamed sediments was performed using standard indices, including D_air_, H_ex_, H_in_, AED_out/in_, ELCR, I_α_, Ra_eq_, and I_γ_ (Table 5). The mean D_air_, a key metric for gamma exposure, was determined to be 40.94 ± 5.16 nGy/h, with individual values ranging from 31.11 to 49.54 nGy/h. This mean is markedly lower than the global average of 59 nGy/h [11,13,73], indicating a negligible radiological impact. Besides the estimated D_air_ values, the AED for outdoor and indoor exposures was calculated using factors (0.7 Sv/Gy) and an occupancy of 0.2 (outdoor) and 0.8 (indoor). Both the resulting average outdoor (AED_out_ = 0.04–0.06 mSv/y) and indoor (AED_in_ = 0.15–0.24 mSv/y) doses fall below established international safety limits [67,69,71]. The potential impact of radiation on human tissues was further assessed using the H_in_ and H_ex_ hazard indices. The mean values calculated for the samples were below the safety threshold of unity, with H_in_ = 0.28 ± 0.03 and H_ex_ = 0.23 ± 0.03 [33,82]. This confirms the absence of significant radiological health risks from the collected sediments [7,32,75].

The index of I_α_ ranged from 0.06 to 0.11, with an average of 0.1 ± 0.02. As this value is well below the safety threshold of 1, it indicates no significant health risk from alpha radiation. Similarly, the I_γ_ values fell between 0.24 and 0.38, averaging 0.32 ± 0.04, which is also below unity. These low indices collectively confirm a minimal radiological health risk associated with the sediments in the Ras Mohamed area [33,72,83]. Furthermore, the Raeq, which represents the combined external and internal dose from gamma rays and alpha particles, was calculated. The mean value of 84.57 ± 11.12 Bq/kg lies safely below the permitted limit of 370 Bq/kg. Finally, the mean ELCR for the beach samples was determined to be 0.18 × 10^−3^ ± 0.03. This is below the recommended reference level of (0.29 × 10^−3^), suggesting that lifetime public exposure to these sediments is unlikely to induce carcinogenic effects [72,76,84,85,86].

### 4.9. Mineralogy

**Figure 10:**
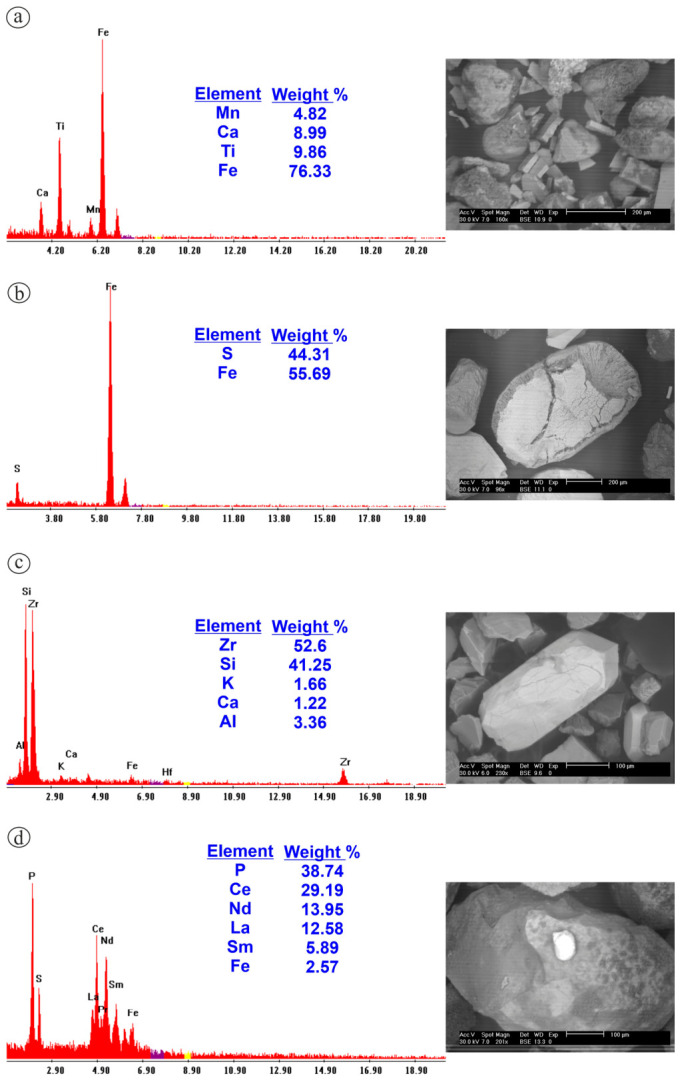
ESEM of the separated minerals: (**a**) magnetite; (**b**) pyrite; (**c**) zircon, and (**d**) monazite.

Heavy minerals were identified using Environmental Scanning Electron Microscopy (ESEM). Samples were first selected with a binocular microscope and then classified. The primary heavy minerals separated were zircon, monazite, magnetite, and pyrite. Magnetite, a ubiquitous iron oxide mineral found in sedimentary, metamorphic, and igneous rocks, often co-occurs with hematite and is a major source of iron ore. In this study, ESEM analysis determined that magnetite grains have a composition dominated by Fe (76.33%), with significant amounts of Ti (9.86%), Ca (8.99%), and Mn (4.82%) (Figure 10a).

Pyrite, the most common sulfide mineral in hydrothermal settings, is frequently associated with Au mineralization [87]. ESEM measurements of the pyrite grains revealed a composition of S (44.31%) and Fe (55.69%) (Figure 10b). Zircon, a widely distributed and common mineral in the Earth’s crust, is prevalent in both sedimentary and igneous rocks, particularly granites [88]. Its exceptional durability and high melting point make it suitable for industrial uses, such as an abrasive and in furnace linings for the steel industry. In the studied stream sediments, zircon constitutes the most abundant non-opaque mineral. ESEM analyses of its euhedral grains identified a composition dominated by Zr (52.6%) and Si (41.25%), with minor amounts of K (1.66%) and Ca (1.22%) (Figure 10c). Monazite, an REE-bearing mineral commonly found in younger granitic rocks, was also identified. ESEM analysis revealed a composition primarily of P (38.74%), Ce (29.19%), and Nd (13.95%), along with significant proportions of La (12.58%), Sm (5.89%), and Fe (2.57%) (Figure 10d).

### 4.10. Strengths and Limitations

This research possesses notable strengths, marking the premier evaluation of metal contamination levels alongside their respective ecological and human health risks within the coastal sediments of Ras Mohamed, located along the northern Red Sea shoreline in South Sinai. However, certain methodological limitations suggest avenues for future inquiry. Fieldwork and sample collection at a depth of 10 cm were confined to a single expedition in June 2024, with sediment sampling restricted primarily to the coastline. Consequently, beach sediments and the eastern maritime expanses extending into the open sea remained unexamined. Because standard screening models rely on total elemental concentrations, the Total Cancer Risk (TCR) calculations omit individual chemical speciation for chromium, lead, and nickel. Lacking specific data to distinguish highly toxic forms—such as Cr^VI^ from Cr^III^—introduces built-in modeling uncertainties that can over- or underestimate true carcinogenic potential. Acknowledging this limitation contextualizes our screening metrics and highlights the critical need for future chemical speciation analysis in protected coastal zones. To build upon these findings, subsequent studies should prioritize high-resolution core and source sampling, utilize isotope geochemistry (e.g., Pb), and investigate seasonal hydrodynamic shifts and biological influences. We acknowledge that using global UCC values may introduce inherent uncertainty as they may misrepresent enrichment due to regional basement geology or carbonate dilution. Moreover, future work should explore effective remediation strategies and broader policy implications.

## 5. Conclusions

This research characterizes the geochemical and radiological profile of the Ras Mohamed coastal zone, establishing a baseline for one of the Red Sea’s most ecologically sensitive regions. The results demonstrate that sediment composition is predominantly influenced by the transport of mineralized materials from western wadi systems and local basement rock weathering. PCA identified five exploratory components (24.09–11.83% variance) linking metal clusters to local lithogenic weathering and tentative anthropogenic influences, though limited sample size necessitates further validation. Ecologically, this study highlights a dichotomy between high accumulation and low immediate toxicity. While the I_geo_ and PLI values indicate moderate enrichment of metals like Cr, V, and Co, the potential ecological risk factors (Er^i^ < 40, and PERI < 150) and mean effects range median quotient (MERMQ = 0.37) indicate that these concentrations are unlikely to cause frequent adverse biological effects. Similarly, the modified hazard quotient (mHQ = 1.64) reflects moderate contamination severity without reaching critical toxicity thresholds. From a human health perspective, the sediments are categorized as safe for recreational and professional exposure. Both non-carcinogenic hazard indices (HIs) and Total Cancer Risk (TCR) estimates for adults and children fall within acceptable regulatory thresholds (HI < 1; TCR < 1 × 10^−4^). Furthermore, the radiological assessment confirms that the natural radioactivity levels of ^226^Ra, ^232^Th, and ^40^K comply with UNSCEAR standards, with the excess lifetime cancer risk (ELCR = 0.18 × 10^−3^) remaining below the world average. Despite these strengths, future monitoring should expand to include seasonal hydrodynamic variations and high-resolution core sampling to track long-term depositional trends. These findings serve as a critical scientific basis for environmental managers and policymakers to balance the blue economy’s growth with the preservation of the Red Sea’s unique coral and mangrove ecosystems.

## Figures and Tables

**Table 1:** Hazard parameters utilized, using Y_Th_, Y_Ra_, and Y_K_ concentrations.

Indices	Equations	Reference
D_air_	D_air_ (nGy/h) = 0.43 Y_Ra_ + 0.666 Y_Th_ + 0.042 Y_K_	[32]
I_γ_	I_γ_ = Y_Ra_/300 + Y_Th_/200 + Y_K_/3000	[32]
Ra_eq_	Ra_eq_ (Bq/kg) = Y_Ra_ + 1.43 Y_Th_ + 0.077 Y_K_	[33]
AED	AED_in_ (mSv/y) = D_air_ × 8.76 ×0.8 (0.2 for outdoor) × 0.7 × 10^−3^	[33]
H_ex_ and H_in_	H_ex_ & H_in_ = Y_Ra_/370 (185 for indoor) + Y_Th_/259 + Y_K_/4810	[33]
I_α_	I_α_ = Y_Ra_/200	[32]
ELCR	AED × DL (70 y) × RF (0.05 Sv^−1^)	[33]

**Table 2:** Descriptive statistics of metal concentrations (mg/kg) in Ras Mohamed sediments, South Sinai.

n = 15	Gravel%	Sand%	Silt%	Clay%	pH	TOM%	Ba	Co	Pb	Cu	Cr	Ni	Zn	V	Fe
**Mean**	5.66	88	6.1	0.2	8.55	0.63	213.13	67.53	34.87	47	136.67	48.47	87.93	201.53	67,347
**Min**	2	82.45	3.6	0.1	7.8	0.44	113	22	17	24	87	25	57	98	56,721
**Max**	10	94.3	8.4	0.3	9.3	0.77	324	99	66	77	232	84	125	278	86,375
**Median**	5	88.15	6.2	0.2	8.5	0.63	245	68	29	45	132	46	87	202	67,072
**SD**	2.7	3.9	1.6	0.06	0.5	0.11	70.92	20.89	13.24	16.71	34.03	15.85	18.01	45.76	6873
**Skewness**	0.15	0.04	−0.24	−0.07	0.2	−0.4	0.06	−0.54	1.08	0.14	1.45	0.56	0.37	−0.48	1.44
**Kurtosis**	−1.37	−1.24	−1.22	−1.02	−0.95	−0.94	−1.48	−0.02	0.81	−1.15	3.81	0.28	0.13	0.44	3.71
**CV**	47.47	4.42	26.2	32.8	5.77	17.46	0.06	−0.54	1.08	0.14	1.45	0.56	0.37	−0.48	1.44
[50]							750	40	70	63	64	50	200	130	−
[40]							668	11.6	17	14.3	35	18.6	52	53	30,890

**Table 3:** Average metal contents (mg/kg) in the Ras Mohamed sediments were compared with corresponding values from Egyptian and international coastal environments, with bold numbers indicating the highest concentrations.

Location	Ba	Co	Pb	Cu	Cr	Ni	Zn	V	Fe	Reference
Ras Mohamed, South Sinai	213.13	**67.53**	34.87	47	136.67	48.47	87.93	**201.53**	**67,347**	This study
El Qulaan, Red Sea, Egypt	994.44	14.22	96.83	55.33	150.83	85	100.94	158.22	49,249	[62]
Abu Ghusun, Egypt	874.43	12	121.9	58.62	152.87	77.5	80.93	160.81	48,927	[55]
Red Sea coastline, Egypt	171.84	4.81	4.89	7.7	53.84	15.37	27.55	29.78	14,562	[53]
W. El-Gemal outlet, Egypt	-	1.24	2.57	0.47	-	2.44	6.74	-	4618	[24]
W. El-Gemal Island, Egypt	-	2.05	0.84	0.31	-	0.7	3.4	-	1271	[54]
Abu Minqar Island, Egypt	-	2.34	1.19	0.27	-	0.76	2.89	-	921	[23]
Marsa Alam coast, Egypt	-	1.83	2.23	1.94	10.62	6.82	28.83	-	1674	[56]
Gulf of Suez, Egypt	-	7.4	2.78	1.66	8.98	5.58	3.96	-	540	[57]
Ras Abu Ali Island, Saudi Arabia	-	1.43	3.5	4.14	7.86	13	6.9	6.67	4808	[58]
Aqaba coast, Saudi Arabia	-	4.5	6.6	30	39	14	24	-	3374	[52]
Lagoon lakes, Turkey	-	-	20.39	48.97	154.36	130.64	75.33	-	48,580	[59]
Mahshahr Bay, Iran	56	3	-	22	43	62	93	-	-	[26]
Northeast coast, Iran	142	8	9	13	70	50	34	41	21,800	[20]
Vedaranyam coast, India	-	71	-	115.1	48.8	66	623	-	65,966	[61]
Zhejiang coast, China	457.4	17.17	29.4	28.15	55.46	45	115.87	142.8	-	[22]
Mailiao coast, Taiwan	-	-	21.69	30.96	86.1	51.65	174.12	-	38,370	[60]

**Table 4:** PCA analysis with eigenvalues, variance, and loadings for the studied metals in Ras Mohamed sediments.

PC	Eigenvalue	Variance %	Metals	PC 1	PC 2	PC 3	PC 4	PC 5
1	2.16	24.09	Ba	0.11	**0.47**	−0.53	−0.13	0.11
2	2.07	23.08	Pb	0.27	−0.02	**0.42**	−0.55	0.39
3	1.34	14.91	Co	−0.41	**0.32**	0.14	−0.20	−0.35
4	1.27	14.17	Cu	−0.13	−0.20	0.32	**0.57**	0.45
5	1.06	11.83	Cr	−0.40	**0.44**	0.00	0.30	0.20
		**88.11**	Ni	**0.58**	0.23	0.06	0.10	0.02
			Zn	0.12	−0.35	**−0.62**	0.12	0.18
			V	**0.39**	0.02	0.17	0.40	**−0.60**
			Fe	0.26	**0.51**	0.07	0.20	0.28

**Table 5:** Measured activity concentrations and corresponding health risk indices.

Sites	^232^Th (Bq/kg)	^226^Ra (Bq/kg)	^40^K (Bq/kg)	Total	^232^Th /^40^K	^226^Th /^40^K	^226^Ra /^226^Th	D_air_ nGy/h	H_in_	H_ex_	I_α_	I_γ_	AED_out_ (mSv/y)	AED_in_ (mSv/y)	Ra_eq_	ELCR
**RM 1**	24.24	22.20	369.34	415.78	0.06	0.07	0.92	41.20	0.29	0.23	0.11	0.32	0.05	0.20	85.30	0.18
**RM 2**	28.28	22.20	406.90	457.38	0.05	0.07	0.79	45.47	0.31	0.25	0.11	0.35	0.06	0.22	93.97	0.20
**RM 3**	12.12	22.20	438.20	472.52	0.05	0.03	1.83	36.02	0.26	0.20	0.11	0.28	0.04	0.18	73.27	0.15
**RM 4**	36.36	22.20	375.60	434.16	0.06	0.10	0.61	49.54	0.34	0.28	0.11	0.38	0.06	0.24	103.12	0.21
**RM 5**	32.32	22.20	331.78	386.30	0.07	0.10	0.69	45.01	0.31	0.25	0.11	0.35	0.06	0.22	93.96	0.19
**RM 6**	20.20	22.20	406.90	449.30	0.05	0.05	1.10	40.09	0.28	0.22	0.11	0.31	0.05	0.20	82.42	0.17
**RM 7**	16.16	22.20	334.91	373.27	0.07	0.05	1.37	34.37	0.25	0.19	0.11	0.27	0.04	0.17	71.10	0.15
**RM 8**	36.36	11.10	349.31	396.77	0.03	0.10	0.31	43.66	0.27	0.24	0.06	0.34	0.05	0.21	89.99	0.19
**RM 9**	32.32	22.20	325.52	380.04	0.07	0.10	0.69	44.74	0.31	0.25	0.11	0.34	0.05	0.22	93.48	0.19
**RM 10**	20.20	22.20	356.82	399.22	0.06	0.06	1.10	37.99	0.27	0.21	0.11	0.29	0.05	0.19	78.56	0.16
**RM 11**	16.16	22.20	349.31	387.67	0.06	0.05	1.37	34.98	0.26	0.20	0.11	0.27	0.04	0.17	72.21	0.15
**RM 12**	36.36	22.20	350.25	408.81	0.06	0.10	0.61	48.47	0.33	0.27	0.11	0.37	0.06	0.24	101.16	0.21
**RM 13**	32.32	11.10	349.93	393.35	0.03	0.09	0.34	41.00	0.26	0.23	0.06	0.32	0.05	0.20	84.26	0.18
**RM 14**	20.20	11.10	306.74	338.04	0.04	0.07	0.55	31.11	0.20	0.17	0.06	0.24	0.04	0.15	63.60	0.13
**RM 15**	16.16	22.20	478.89	517.25	0.05	0.03	1.37	40.42	0.28	0.22	0.11	0.31	0.05	0.20	82.18	0.17
**Min**	12.12	11.10	306.74	338.04	0.03	0.03	0.31	31.11	0.20	0.17	0.06	0.24	0.04	0.15	63.60	0.13
**Max**	36.36	22.20	478.89	517.25	0.07	0.10	1.83	49.54	0.34	0.28	0.11	0.38	0.06	0.24	103.12	0.21
**Mean**	25.32	19.98	368.69	413.99	0.05	0.07	0.91	40.94	0.28	0.23	0.10	0.32	0.05	0.20	84.57	0.18
**SD**	8.28	4.44	44.52	43.69	0.01	0.03	0.42	5.16	0.03	0.03	0.02	0.04	0.01	0.03	11.12	0.02

**Table 6:** Comparison of the measured radionuclide concentrations with data from other studies.

Location	^226^Ra (Bq/kg)	^232^Th (Bq/kg)	^40^K (Bq/kg)	References
Ghadir soil, Egypt	20.02	32.07	289.31	[78]
Abu Ghusun, Egypt	25.43	29.55	337.06	[55]
Qulaan soil, Egypt	25.43	19.99	294.92	[62]
Sharm El Luli, Egypt	24.57	23.32	241.83	[77]
Saudi Arabia	6–54	7–52	299–761	[79]
Aegean sediment, Greece	9–31	9–67	426–740	[2]
Konya sediment, Turkey	28.24	29.7	366.03	[68]
Shoreline sediment, Thailand	2.7–23.5	3.0–31.2	10.7–654.3	[80]
Dois Rios sediment, Brazil	39	48	412	[16]
Leepa Valley	28.5	48.4	666.7	[81]
Jeddah soil	13.14	5.05	139.09	[12]
—	33	45	412	[33]
—	19.98	25.32	368.69	This study

## Data Availability

The original contributions presented in this study are included in the article/Supplementary Materials. Further inquiries can be directed to the corresponding authors.

## Supplementary Materials

The following supporting information can be downloaded at https://www.mdpi.com/article/10.3390/toxics14060464/s1. Figure S1: Gamma-ray spectrometry utilizing multi-channel analyzers; Table S1: The classification and description of ecological and sediment quality guidelines (SQGs) and human health risk indices utilized to assess the heavy metals in Ras Mohamed coastal sediments, South Sinai; Table S2: The values of input parameters to estimate chronic daily intake (CDI); Table S3: The pH, total organic matter, grain size analysis, and sediment type of Ras Mohamed coastal sediments, north Red Sea, South Sinai; Table S4: The analyzed heavy metal contents (mg/kg) in Ras Mohamed coastal sediments, South Sinai; Table S5: The enrichment factor (EF) of the metals investigated in Ras Mohamed coastal sediments, South Sinai; Table S6:The contamination factor (CF), and pollution load index (PLI) of heavy metals in Ras Mohamed coastal sediments, South Sinai; Table S7: The geo-accumulation factor (Igeo) of heavy metals in Ras Mohamed coastal sediments, South Sinai; Table S8: Potential ecological risk indices Eri and PERI for heavy metals in Ras Mohamed coastal sediments, South Sinai; Table S9: The SQG mean effects range median quotient (MERMQ) for heavy metals in Ras Mohamed coastal sediments, South Sinai; Table S10: The SQG toxic risk index (TRI) for heavy metals in Ras Mohamed coastal sediments, South Sinai; Table S11: The SQG modified hazard quotient (mHQ) for heavy metals in Ras Mohamed coastal sediments, South Sinai; Table S12: Human health non-carcinogenic risk index (HQ) for heavy metals in Ras Mohamed coastal sediments, South Sinai; Table S13: Human health carcinogenic risk index (TCR) for heavy metals in Ras Mohamed coastal sediments, South Sinai.
