# Supplementary material for: Trace Metal Enrichment and Radiological Risk in Coastal Sediments: Implications for Ecological and Human Health Safety
Source: Toxics. 2026 May 26;14(6):464. doi: 10.3390/toxics14060464 (PMC13306867; doi:10.3390/toxics14060464)
Supplement: Supplementary file 1 [file toxics-14-00464-s001.zip › toxics-4324632-supplementary.pdf]

toxics

Trace Metal Enrichment and Radiological Risk in Coastal Sediments: Implications for Ecological and Human Health Safety

El Saeed R. Lasheen [1,*], Tamader Alhazani [2], Gehad M. Saleh [3], Basma A. El-Badry [2], Mabrouk Sami [4,*], Ioan V. Sanislav [5], and Ahmed Abdelaal [6]

1 Geology Department, Faculty of Science, Al-Azhar University, Cairo 11884, Egypt
2 Physics Department, Faculty of Science, Imam Mohammad Ibn Saud Islamic University (IMSIU), Riyadh 11432, Saudi Arabia; tmalhazani@imam.edu.sa (T.A.); baabdulladeem@imamu.edu.sa (B.A.E.-B.)
3 Nuclear Materials Authority, Cairo, Egypt; drgehad_m@yahoo.com
4 Geosciences Department, College of Science, United Arab Emirates University, Al Ain 15551, United Arab Emirates
5 Economic Geology Research Centre (EGRU), College of Science and Engineering, James Cook University, Townsville, QLD 4811, Australia; ioan.sanislav@jcu.edu.au (I.V.S.)
6 Environmental Sciences Department, Faculty of Science, Port Said University, Port Said 42522, Egypt; ahmed_abdelaal@sci.psu.edu.eg
* Correspondence: elsaeedlasheen@azhar.edu.eg (E.S.R.L.); mabrouk.hassan@mu.edu.eg (M.S.)

Methodology

The quantification of uranium and thorium activity concentrations in ore and lithological samples predominantly depends on precise radiometric techniques, notably through the deployment of gamma-ray spectrometry utilizing multi-channel analyzers (Figure 1). In the present investigation, radiometric analyses were performed using NaI (Tl) scintillation detectors sourced from GeoMetrics, located in San Jose, CA, USA, operated within the Radiation Protection Department laboratory of the Nuclear and Radiological Regulatory Authority (Egypt). The NaI (Tl) scintillation detector, while exhibiting relatively limited energy resolution, is distinguished by its superior detection efficiency. This high intrinsic efficiency facilitates the rapid and accurate determination of radionuclide activity concentrations of ^{238}U, ^{232}Th, and ^{40}K in diverse geological matrices, including rocks, soils, and sand samples. The results depend on the accuracy of the energy calibration procedure, which takes into account the possibility of interference of the individual nuclides within each peak region. The gamma-ray spectrometry system comprises a Bicron scintillation detector, manufactured by Bicron in Newbury, OH, USA, equipped with a 76 × 76 mm NaI (Tl) crystal hermetically sealed and coupled to a photomultiplier tube housed in an aluminum casing. The detector is protected from ambient radiation by a chamber made of lead bricks and from induced X-rays by a cylindrical copper shield (0.6 cm thick). A 5 cm thick lead cover is then placed over the detector. The detector relates to the Tennelec high-voltage power supply with a digital HV display and a Nuclear Enterprises main shaping amplifier. A laser printer and Nuclease PCA-8000 computerized 8192 multichannel analyzers with a color graphical spectrum display and advanced technical operation features are also connected to the detector.

The measurements were carried out in cylindrical plastic sample containers with a volume of 212.6 cm^3, an average diameter of 9.5 cm, and a height of 3 cm. The measurement of radionuclides is based on the selection of three energy regions of interest (ROIs) representing ^{234}Th, ^{212}Pb, and ^{40}K for U, Th, and K, respectively. It is assumed that the samples are in secular equilibrium, which allows for the indirect determination of the parent radionuclides' activity concentrations through their gamma-emitting progeny.

The gamma transition (92.6 keV) from ^{234}Th decay was used to determine the activity concentration of ^{238}U, while the gamma transitions (352 and 238.6 keV) from the ^{212}Pb decay was used to determine the activity concentrations of ^{232}Th. The activity concentration of ^{40}K was measured directly from the 1460.8 keV peak energy. Uranium and thorium are not gamma emitters, and consequently, they are measured indirectly using their γ-ray-emitting daughters, ^{234}Th and ^{212}Pb, respectively.

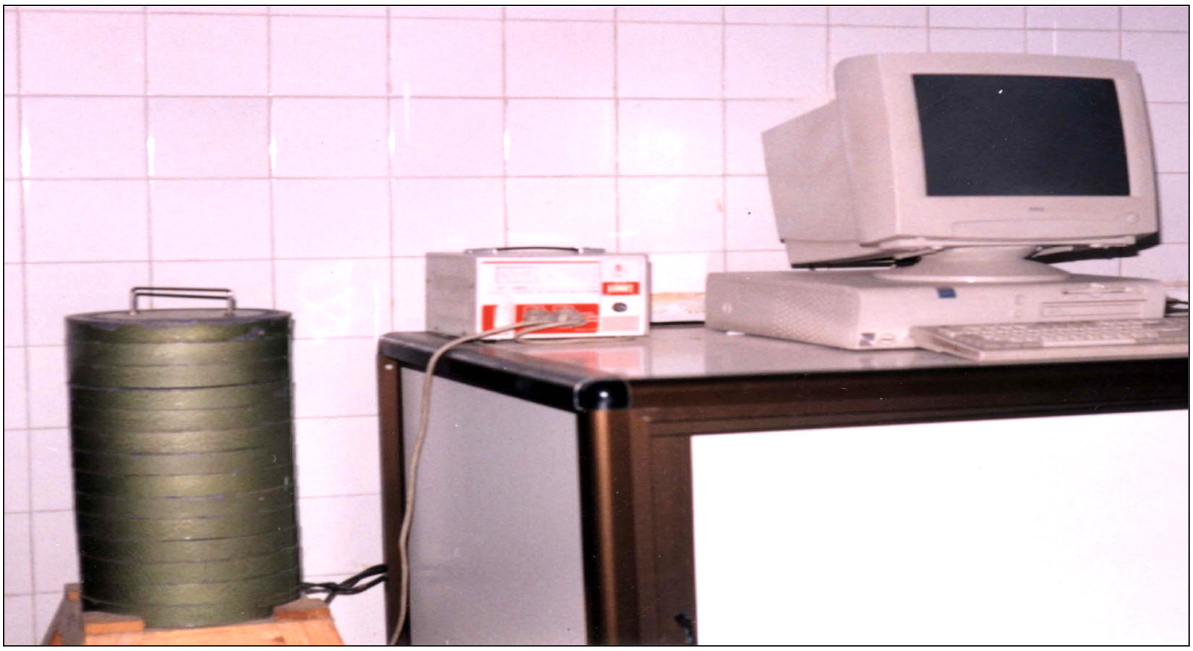

Supplementary Figure 1: NaI(Tl) detector.

Figure 1: Gamma-ray spectrometry utilizing multi-channel analyzers.

The following equation was used to obtain the activity, which was then converted to the activity concentration in Bq/kg for each sample.

$$\mathbf{Ac\ (Bq/kg) = Cn/PGM\varepsilon K}$$

where Ac is the activity concentration for the element in the sample determined by Bq/kg, (Cn) is the net count rate under the corresponding peak, (PG) is the absolute transition probability of the specific gamma ray, (M) is the mass of the sample (kg), Ɛ is the detector efficiency at the specific gamma-ray energy, and (K) is the correction factor for the nuclide decay from the time of sampling to counting. Numerous factors affect the quality of the results obtained,

including the following: (i) the size and energy resolution of the scintillation detector, (ii) geometry and mass of the sample, (iii) performance of the multichannel analyzer and its operation stability, (iv) shielding of the detector, (v) selection of specific detected gammaray energies, (vi) time of measurements, (vii) quality and reliability of the standards used, and (viii) the data processing technique. The process of the radionuclide measurement by the NaI (Tl) detector is carried out after two main steps: (a) energy and (b) sensitivity calibrations.

The permanent calibration was performed with the radioactive calibration sources ^{137}Cs (661.6 keV) and ^{57}Co (122.1 keV) to ensure that the device accurately records the gamma radiation energy of the radioactive elements as follows: Calibration started with the ^{137}Cs source (gain adjustment) and then with the ^{57}Co source (zero adjustment). The ^{137}Cs source was used repeatedly as the minimum procedure.

Sensitivity Calibration: Since the gamma-ray spectrometer is used for geochemical prospecting, it must be calibrated in terms of isotopic sensitivity (i.e., converting counts per unit of time into an isotopic concentration in parts per million or percent). Three synthetic standard sources (geological reference materials) were used for the calibration. These standards were prepared using a series of certified reference samples with specific U, Th, and K concentrations obtained from the International Atomic Energy Agency (IAEA), Vienna, Austria. The sensitivity of the instruments used was determined by measuring the three standards twice, each for 1000 s, and then averaging the gross counts collected in the selected ROIs. The count rates were normalized per unit mass and adjusted for background count rates in the ROIs. The corrected values were entered into the computer program "ANALYSIS", which was developed for laboratory gamma-ray spectrometry of geological materials. The final result is a matrix of instrument sensitivity values, each of which is represented by the count rate per unit mass of the geologic material measured and per unit of the radioelement concentration. These sensitivity values of the equipment are used as a reference in the analysis of the unknown rock samples. The lower limit of detection of ^{238}U, ^{232}Th, and ^{40}K is 44.12, 42.33, and 1028 Bq/kg, respectively. The expected measurement errors usually range between 7 and 12%.

Table S1. The classification and description of ecological and sediment quality guidelines (SQGs) and human health risk indices utilized to assess the heavy metals in Ras Mohamed coastal sediments, South Sinai.

Index	Classification	Description
Enrichment factor (EF)	EF ≤ 1	Background concentration
	EF = 1-2	Deficiency to minimal enrichment
	EF = 2-5	Moderate enrichment
	EF = 5-20	Significant enrichment
	EF = 20-40	Very high enrichment
	EF > 40	Extremely high enrichment
Contamination factor (CF)	CF ≤ 1	Low contamination

	$1 \leq CF < 3$	Moderate contamination
	$3 \leq CF < 6$	Considerable contamination
	$CF \geq 6$	High contamination
Index of geo-accumulation (I_{geo})	$I_{geo} = 0$	Uncontaminated sediments
	$0 < I_{geo} \leq 1$	Uncontaminated to moderately contaminated
	$1 < I_{geo} \leq 2$	Moderately contaminated
	$2 < I_{geo} \leq 3$	Moderately to strongly contaminated
	$3 < I_{geo} \leq 4$	Strongly contaminated
	$4 < I_{geo} \leq 5$	Strongly to extremely contaminated
	$I_{geo} > 5$	Extremely contaminated
Pollution load index (PLI)	PLI = 0	No pollution
	PLI = 1	Baseline levels of contamination
	PLI >1	Progressive contamination
Potential ecological risk index (PERI)	PERI < 150	Low potential ecological risk
	150 ≤ PERI < 300	Moderate ecological risk
	300 ≤ PERI < 600	Severe ecological risk
	PERI > 600	Serious ecological risk
Potential ecological risk index (Er^i)	$Er^i < 40$	Low
	$40 \leq Er^i < 80$	Moderate
	$80 \leq Er^i < 160$	Considerable
	$160 \leq Er^i < 320$	High
	$Er^i \geq 320$	Very high
Mean effects range median quotient (MERMQ)	MERMQ ≤ 0.1	Low-priority risk level
	0.11 < MERMQ ≤ 0.5	Low–medium-priority risk level
	0.51< MERMQ ≤ 1.5	Medium–high-priority risk level
	MERMQ ≥ 1.5	High-priority risk level
Toxic risk index (TRI)	TRI ≤ 5	No toxic risk
	5 < TRI ≤ 10	Low toxic risk
	10 < TRI ≤ 15	Moderate toxic risk
	15 < TRI ≤ 20	Considerable toxic risk
	TRI > 20	Very high toxic risk
Modified hazard quotient (mHQ)	mHQ < 0.5	Nil to very low severity of contamination
	0.5 ≤ mHQ < 1.0	Very low severity of contamination
	1.0 ≤ mHQ < 1.5	Low severity of contamination
	1.5 ≤ mHQ < 2.0	Moderate severity of contamination
	2.0 ≤ mHQ < 2.5	Considerable severity of contamination
	2.5 ≤ mHQ < 3.0	High severity of contamination
	3.0 ≤ mHQ < 3.5	Very high severity of contamination
	mHQ > 3.5	Extreme severity of contamination
Hazard quotient (HQ)	HQ < 1	Adverse health effects would unlikely occur
	HQ > 1	Potential non-carcinogenic effects would occur

Hazard index (HI)	HI < 1	No significant non-carcinogenic risk
	Hi > 1	Adverse non-carcinogenic risk effects may occur

Table S2. The values of input parameters to estimate chronic daily intake (CDI).

Parameter	CDI (Children)	CDI (Adult)
CF	1×10^{-6}	
SA	2800 cm^2	5700 cm^2
AF	0.2	0.07
ABS	0.001	
EF	1 events/day × 350 days/year	
ED	6 years	30 years
BW	15 kg	70 kg
AT	Non-carcinogenic: ED × 365 days/years; carcinogenic: 70 years × 365 days/years	

Table S3. The pH, total organic matter, grain size analysis, and sediment type of Ras Mohamed coastal sediments, north Red Sea, South Sinai.

Sample	pH	TOM%	Gravel%	Sand%	Silt%	Clay%	Sediment type
RM1	9.3	0.77	2	94.3	3.6	0.1	silty sand
RM2	9.2	0.75	3	92.6	4.2	0.2	silty sand
RM3	9.3	0.76	5	89.4	5.5	0.1	silty sand
RM4	9.1	0.74	2	91.6	6.2	0.2	silty sand
RM5	8.7	0.73	3	93.05	3.8	0.15	silty sand
RM6	8.5	0.68	4	90.1	5.6	0.3	silty sand
RM7	8.4	0.65	7	86.95	5.8	0.25	gravely silty sand
RM8	8.6	0.63	6	89.6	4.2	0.2	gravely silty sand
RM9	8.5	0.61	8	84.9	6.8	0.3	gravely silty sand
RM10	8.4	0.62	4	88.15	7.7	0.15	silty sand
RM11	8.5	0.58	5	86.65	8.1	0.25	silty Sand
RM12	8.1	0.51	9	82.45	8.4	0.15	gravely silty sand
RM13	8	0.54	8	84.2	7.6	0.2	gravely silty sand
RM14	7.9	0.45	9	83.95	6.8	0.25	gravely silty sand
RM15	7.8	0.44	10	82.5	7.2	0.3	gravely silty sand

Table S4. The analyzed heavy metal contents (mg/kg) in Ras Mohamed coastal sediments, South Sinai.

Sample	Ba	Co	Pb	Cu	Cr	Ni	Zn	V	Fe
RM1	245	68	29	66	157	25	125	98	67,072
RM2	259	47	66	33	163	34	86	165	64,694
RM3	315	62	25	24	147	62	97	174	67,771
RM4	277	45	38	65	232	54	57	195	75,395
RM5	324	99	48	29	122	46	69	177	70.919
RM6	247	87	24	45	132	84	87	245	86,375
RM7	269	82	36	27	98	67	92	236	67,072
RM8	178	67	17	77	87	55	102	214	62,176
RM9	245	22	27	44	114	52	115	278	63.785
RM10	149	73	26	41	128	46	86	251	65,323
RM11	156	85	38	57	135	43	97	233	69,101
RM12	113	78	41	53	129	33	77	202	67,772
RM13	156	49	55	62	158	38	69	226	65,953
RM14	142	57	26	27	136	29	88	165	60,078
RM15	122	92	27	55	112	59	72	164	56,721

https://doi.org/10.3390/xxxxx

Table S5. The enrichment factor (EF) of the metals investigated in Ras Mohamed coastal sediments, South Sinai.

Samples	Ba	Co	Pb	Cu	Cu	Ni	Zn	V
RM1	0.17	2.70	0.79	2.13	2.07	0.62	1.11	0.75
RM2	0.19	1.93	1.85	1.10	2.22	0.87	0.79	1.31
RM3	0.21	2.44	0.67	0.76	1.91	1.52	0.85	1.32
RM4	0.17	1.59	0.92	1.86	2.72	1.19	0.45	1.33
RM5	0.21	3.72	1.23	0.88	1.52	1.08	0.58	1.28
RM6	0.13	2.68	0.50	1.13	1.35	1.62	0.60	1.46
RM7	0.19	3.26	0.98	0.87	1.29	1.66	0.81	1.81
RM8	0.13	2.87	0.50	2.68	1.23	1.47	0.97	1.77
RM9	0.18	0.92	0.77	1.49	1.58	1.35	1.07	2.24
RM10	0.11	2.98	0.72	1.36	1.73	1.17	0.78	1.98
RM11	0.10	3.28	1.00	1.78	1.72	1.03	0.83	1.74
RM12	0.08	3.06	1.10	1.69	1.68	0.81	0.67	1.53
RM13	0.11	1.98	1.52	2.03	2.11	0.96	0.62	1.76
RM14	0.11	2.53	0.79	0.97	2.00	0.80	0.87	1.41
RM15	0.10	0.08	0.86	2.09	1.74	1.73	0.75	1.49

Table S6. The contamination factor (CF), and pollution load index (PLI) of heavy metals in Ras Mohamed coastal sediments, South Sinai.

Samples	CF									**PLI**
	Ba	Co	Pb	Cu	Cr	Ni	Zn	V	Fe	
RM1	0.37	5.86	1.71	4.62	4.49	1.34	2.40	1.63	2.17	**2.12**
RM2	0.39	4.05	3.88	2.31	4.66	1.83	1.65	2.75	2.09	**2.19**
RM3	0.47	5.34	1.47	1.68	4.20	3.33	1.87	2.90	2.19	**2.16**
RM4	0.41	3.88	2.24	4.55	6.63	2.90	1.10	3.25	2.44	**2.41**
RM5	0.49	8.53	2.82	2.03	3.49	2.47	1.33	2.95	2.30	**2.30**
RM6	0.37	7.50	1.41	3.15	3.77	4.52	1.67	4.08	2.80	**2.51**
RM7	0.40	7.07	2.12	1.89	2.80	3.60	1.77	3.93	2.17	**2.29**
RM8	0.27	5.78	1.00	5.38	2.49	2.96	1.96	3.57	2.01	**2.12**
RM9	0.37	1.90	1.59	3.08	3.26	2.80	2.21	4.63	2.06	**2.06**
RM10	0.22	6.29	1.53	2.87	3.66	2.47	1.65	4.18	2.11	**2.11**
RM11	0.23	7.33	2.24	3.99	3.86	2.31	1.87	3.88	2.24	**2.35**
RM12	0.17	6.72	2.41	3.71	3.69	1.77	1.48	3.37	2.19	**2.08**
RM13	0.23	4.22	3.24	4.34	4.51	2.04	1.33	3.77	2.14	**2.23**
RM14	0.21	4.91	1.53	1.89	3.89	1.56	1.69	2.75	1.94	**1.76**
RM15	0.18	7.93	1.59	3.85	3.20	3.17	1.38	2.73	1.84	**2.04**

Table S7. The geo-accumulation factor (I_{geo}) of heavy metals in Ras Mohamed coastal sediments, South Sinai.

Samples	Ba	Co	Pb	Cu	Cr	Ni	Zn	V	Fe
RM1	16.74	9.04	8.36	9.30	11.84	8.28	12.08	11.94	30.36
RM2	16.82	8.51	9.55	8.30	11.89	8.72	11.54	12.69	30.31
RM3	17.10	8.91	8.15	7.84	11.74	9.59	11.72	12.76	30.38
RM4	16.91	8.44	8.75	9.28	12.40	9.39	10.95	12.93	30.53
RM5	17.14	9.58	9.09	8.11	11.48	9.16	11.22	12.79	30.44
RM6	16.75	9.39	8.09	8.74	11.59	10.02	11.56	13.26	30.73
RM7	16.87	9.31	8.67	8.01	11.16	9.70	11.64	13.20	30.36
RM8	16.27	9.02	7.59	9.52	10.99	9.41	11.79	13.06	30.25
RM9	16.74	7.41	8.26	8.71	11.38	9.33	11.96	13.44	30.29
RM10	16.02	9.14	8.20	8.61	11.54	9.16	11.54	13.29	30.33
RM11	16.08	9.91	8.75	9.09	11.62	9.06	11.72	13.19	30.41
RM12	15.62	9.54	8.86	8.98	11.56	8.68	11.38	12.98	30.38
RM13	16.08	10.16	9.28	9.21	11.85	8.88	11.22	13.14	30.34
RM14	15.95	9.47	8.20	8.01	11.63	8.49	11.57	12.69	30.20
RM15	15.73	11.64	8.26	9.03	11.35	9.51	11.29	12.68	30.12

Table S8. Potential ecological risk indices Er^i and PERI for heavy metals in Ras Mohamed coastal sediments, South Sinai.

Samples	Er^i								**PERI**	**Risk Grade**
	Co	Pb	Cu	Cr	Ni	Zn	V	Fe		
RM1	29.31	8.53	23.08	8.97	6.72	2.40	3.27	2.17	**84.45**	Low
RM2	20.26	19.41	11.54	9.31	9.14	1.65	5.50	2.09	**78.91**	Low
RM3	26.72	7.35	8.39	8.40	16.67	1.87	5.80	2.19	**77.39**	Low
RM4	19.40	11.18	22.73	13.26	14.52	1.10	6.50	2.44	**91.11**	Low
RM5	42.67	14.12	10.14	6.97	12.37	1.33	5.90	2.30	**95.79**	Low
RM6	37.50	7.06	15.73	7.54	22.58	1.67	8.17	2.80	**103.05**	Low
RM7	35.34	10.59	9.44	5.60	18.01	1.77	7.87	2.17	**90.79**	Low
RM8	28.88	5.00	26.92	4.97	14.78	1.96	7.13	2.01	**91.67**	Low
RM9	9.48	7.94	15.38	6.51	13.98	2.21	9.27	2.06	**66.84**	Low
RM10	31.47	7.65	14.34	7.31	12.37	1.65	8.37	2.11	**85.26**	Low
RM11	36.64	11.18	19.93	7.71	11.56	1.87	7.77	2.24	**98.89**	Low
RM12	33.62	12.06	18.53	7.37	8.87	1.48	6.73	2.19	**90.86**	Low
RM13	21.12	16.18	21.68	9.03	10.22	1.33	7.53	2.14	**89.21**	Low
RM14	24.57	7.65	9.44	7.77	7.80	1.69	5.50	1.94	**66.36**	Low
RM15	39.66	7.94	19.23	6.40	15.86	1.38	5.47	1.84	**97.77**	Low
**Mean**	**29.11**	**10.25**	**16.43**	**7.81**	**13.03**	**1.69**	**6.72**	**2.18**		
**Er^i grade**	Low	Low	Low	Low	Low	Low	Low	Low		

Table S9. The SQG mean effects range median quotient (MERMQ) for heavy metals in Ras Mohamed coastal sediments, South Sinai.

Samples	Pb	Cu	Cr	Ni	Zn	**MERMQ**
RM1	0.13	0.24	0.42	0.48	0.30	**0.32**
RM2	0.30	0.12	0.44	0.66	0.21	**0.35**
RM3	0.11	0.09	0.40	1.20	0.24	**0.41**
RM4	0.17	0.24	0.63	1.05	0.14	**0.45**
RM5	0.22	0.11	0.33	0.89	0.17	**0.34**
RM6	0.11	0.17	0.36	1.63	0.21	**0.49**
RM7	0.17	0.10	0.26	1.30	0.22	**0.41**
RM8	0.08	0.29	0.24	1.07	0.25	**0.38**
RM9	0.12	0.16	0.31	1.01	0.28	**0.38**
RM10	0.12	0.15	0.35	0.89	0.21	**0.34**
RM11	0.17	0.21	0.36	0.83	0.24	**0.36**
RM12	0.19	0.20	0.35	0.64	0.19	**0.31**
RM13	0.25	0.23	0.43	0.74	0.17	**0.36**
RM14	0.12	0.10	0.37	0.56	0.21	**0.27**
RM15	0.12	0.20	0.30	1.14	0.18	**0.39**

Table S10. The SQG toxic risk index (TRI) for heavy metals in Ras Mohamed coastal sediments, South Sinai.

Samples	Pb	Cu	Cr	Ni	Zn	**TRI**
RM1	1.17	0.10	5.21	2.85	0.42	**6.47**
RM2	2.64	0.20	2.57	4.30	0.46	**6.65**
RM3	2.34	0.28	3.45	3.82	0.34	**6.35**
RM4	2.42	0.38	2.01	4.17	0.26	**8.53**
RM5	3.79	0.44	1.58	5.31	0.48	**5.78**
RM6	1.36	0.54	0.92	6.37	0.22	**7.24**
RM7	1.90	0.46	1.42	5.97	0.26	**5.93**
RM8	1.56	2.46	1.79	1.01	0.24	**6.14**
RM9	2.08	1.05	1.50	3.29	0.60	**6.04**
RM10	1.84	0.99	2.65	2.55	1.23	**5.81**
RM11	3.07	0.74	3.22	2.94	1.32	**6.51**
RM12	3.35	2.64	2.85	1.71	1.02	**5.91**
RM13	3.14	2.19	3.04	1.58	0.67	**7.03**
RM14	0.84	1.19	3.14	3.82	0.72	**5.06**
RM15	2.03	1.37	5.87	4.17	0.78	**6.23**

Table S11. The SQG modified hazard quotient (mHQ) for heavy metals in Ras Mohamed coastal sediments, South Sinai.

Samples	Pb	Cu	Cr	Ni	Zn
RM1	1.07	1.77	2.54	1.40	1.21
RM2	1.62	1.25	2.59	1.63	1.00
RM3	1.00	1.07	2.46	2.20	1.06
RM4	1.23	1.76	3.09	2.05	0.82
RM5	1.38	1.17	2.24	1.89	0.90
RM6	0.98	1.46	2.33	2.56	1.01
RM7	1.20	1.13	2.01	2.29	1.04
RM8	0.82	1.91	1.89	2.07	1.09
RM9	1.04	1.44	2.17	2.01	1.16
RM10	1.02	1.39	2.29	1.89	1.00
RM11	1.23	1.64	2.36	1.83	1.06
RM12	1.28	1.59	2.30	1.60	0.95
RM13	1.48	1.72	2.55	1.72	0.90
RM14	1.02	1.13	2.37	1.50	1.01
RM15	1.04	1.62	2.15	2.14	0.92

Table S12. Human health non-carcinogenic risk index (HQ) for heavy metals in Ras Mohamed coastal sediments, South Sinai.

Samples	Co	Pb	Cu	Cr	Ni	Zn	**HI**
	HQ Child						
RM1	7.30E-04	1.78E-03	3.82E-04	2.25E-03	2.68E-04	8.95E-05	**5.50E-03**
RM2	5.05E-04	4.05E-03	1.91E-04	2.33E-03	3.65E-04	6.16E-05	**7.51E-03**
RM3	6.66E-04	1.53E-03	1.39E-04	2.10E-03	6.66E-04	6.95E-05	**5.18E-03**
RM4	4.83E-04	2.33E-03	3.76E-04	3.32E-03	5.80E-04	4.08E-05	**7.13E-03**
RM5	1.06E-03	2.95E-03	1.68E-04	1.75E-03	4.94E-04	4.94E-05	**6.47E-03**
RM6	9.34E-04	1.47E-03	2.61E-04	1.89E-03	9.02E-04	6.23E-05	**5.52E-03**
RM7	8.81E-04	2.21E-03	1.56E-04	1.40E-03	7.20E-04	6.59E-05	**5.44E-03**
RM8	7.20E-04	1.04E-03	4.46E-04	1.25E-03	5.91E-04	7.30E-05	**4.12E-03**
RM9	2.36E-04	1.66E-03	2.55E-04	1.63E-03	5.58E-04	8.23E-05	**4.42E-03**
RM10	7.84E-04	1.60E-03	2.37E-04	1.83E-03	4.94E-04	6.16E-05	**5.01E-03**
RM11	9.13E-04	2.33E-03	3.30E-04	1.93E-03	4.62E-04	6.95E-05	**6.04E-03**
RM12	8.38E-04	2.52E-03	3.07E-04	1.85E-03	3.54E-04	5.51E-05	**5.92E-03**
RM13	5.26E-04	3.38E-03	3.59E-04	2.26E-03	4.08E-04	4.94E-05	**6.98E-03**
RM14	6.12E-04	1.60E-03	1.56E-04	1.95E-03	3.11E-04	6.30E-05	**4.69E-03**
RM15	9.88E-04	1.66E-03	3.18E-04	1.60E-03	6.34E-04	5.16E-05	**5.25E-03**
	HQ Adult						
RM1	5.58E-04	1.36E-03	2.92E-04	1.72E-03	2.05E-04	6.83E-05	**4.20E-03**
RM2	3.85E-04	3.09E-03	1.46E-04	1.78E-03	2.79E-04	4.70E-05	**5.73E-03**
RM3	5.08E-04	1.17E-03	1.06E-04	1.61E-03	5.08E-04	5.30E-05	**3.95E-03**
RM4	3.69E-04	1.78E-03	2.87E-04	2.54E-03	4.43E-04	3.12E-05	**5.45E-03**
RM5	8.12E-04	2.25E-03	1.28E-04	1.33E-03	3.77E-04	3.77E-05	**4.94E-03**
RM6	7.13E-04	1.12E-03	1.99E-04	1.44E-03	6.89E-04	4.76E-05	**4.22E-03**
RM7	6.72E-04	1.69E-03	1.19E-04	1.07E-03	5.49E-04	5.03E-05	**4.15E-03**
RM8	5.49E-04	7.96E-04	3.40E-04	9.51E-04	4.51E-04	5.58E-05	**3.14E-03**
RM9	1.80E-04	1.26E-03	1.94E-04	1.25E-03	4.26E-04	6.29E-05	**3.38E-03**
RM10	5.99E-04	1.22E-03	1.81E-04	1.40E-03	3.77E-04	4.70E-05	**3.82E-03**
RM11	6.97E-04	1.78E-03	2.52E-04	1.48E-03	3.53E-04	5.30E-05	**4.61E-03**
RM12	6.39E-04	1.92E-03	2.34E-04	1.41E-03	2.71E-04	4.21E-05	**4.52E-03**
RM13	4.02E-04	2.58E-03	2.74E-04	1.73E-03	3.12E-04	3.77E-05	**5.33E-03**
RM14	4.67E-04	1.22E-03	1.19E-04	1.49E-03	2.38E-04	4.81E-05	**3.58E-03**
RM15	7.54E-04	1.26E-03	2.43E-04	1.22E-03	4.84E-04	3.94E-05	**4.01E-03**

Table S13. Human health carcinogenic risk index (TCR) for heavy metals in Ras Mohamed coastal sediments, South Sinai.

Samples	Pb	Cr	Ni	**TCR**
	CR Child			
RM1	2.24E-08	5.78E-05	4.19E-07	**5.83E-05**
RM2	5.10E-08	6.00E-05	5.70E-07	**6.06E-05**
RM3	1.93E-08	5.41E-05	1.04E-06	**5.52E-05**
RM4	2.94E-08	8.54E-05	9.05E-07	**8.64E-05**
RM5	3.71E-08	4.49E-05	7.71E-07	**4.57E-05**
RM6	1.86E-08	4.86E-05	1.41E-06	**5.00E-05**
RM7	2.78E-08	3.61E-05	1.12E-06	**3.72E-05**
RM8	1.31E-08	3.20E-05	9.21E-07	**3.30E-05**
RM9	2.09E-08	4.20E-05	8.71E-07	**4.29E-05**
RM10	2.01E-08	4.71E-05	7.71E-07	**4.79E-05**
RM11	2.94E-08	4.97E-05	7.20E-07	**5.05E-05**
RM12	3.17E-08	4.75E-05	5.53E-07	**4.81E-05**
RM13	4.25E-08	5.82E-05	6.37E-07	**5.89E-05**
RM14	2.01E-08	5.01E-05	4.86E-07	**5.06E-05**
RM15	2.09E-08	4.12E-05	9.88E-07	**4.22E-05**
	CR Adult			
RM1	8.56E-08	2.21E-04	1.60E-06	**2.22E-04**
RM2	1.95E-07	2.29E-04	2.17E-06	**2.31E-04**
RM3	7.38E-08	2.07E-04	3.96E-06	**2.11E-04**
RM4	1.12E-07	3.26E-04	3.45E-06	**3.30E-04**
RM5	1.42E-07	1.71E-04	2.94E-06	**1.75E-04**
RM6	7.08E-08	1.86E-04	5.37E-06	**1.91E-04**
RM7	1.06E-07	1.38E-04	4.28E-06	**1.42E-04**
RM8	5.02E-08	1.22E-04	3.52E-06	**1.26E-04**
RM9	7.97E-08	1.60E-04	3.33E-06	**1.64E-04**
RM10	7.67E-08	1.80E-04	2.94E-06	**1.83E-04**
RM11	1.12E-07	1.90E-04	2.75E-06	**1.93E-04**
RM12	1.21E-07	1.81E-04	2.11E-06	**1.84E-04**
RM13	1.62E-07	2.22E-04	2.43E-06	**2.25E-04**
RM14	7.67E-08	1.91E-04	1.85E-06	**1.93E-04**
RM15	7.97E-08	1.57E-04	3.77E-06	**1.61E-04**
